# Information theoretic measures of causal influences during transient neural events

**DOI:** 10.3389/fnetp.2023.1085347

**Published:** 2023-05-31

**Authors:** Kaidi Shao, Nikos K. Logothetis, Michel Besserve

**Affiliations:** ^1^ International Center for Primate Brain Research (ICPBR), Center for Excellence in Brain Science and Intelligence Technology (CEBSIT), Chinese Academy of Sciences (CAS), Shanghai, China; ^2^ Department of Cognitive Neurophysiology, Max Planck Institute for Biological Cybernetics, Tübingen, Germany; ^3^ Graduate School of Neural and Behavioral Sciences, International Max Planck Research School, Eberhard-Karls University of Tübingen, Tübingen, Germany; ^4^ Centre for Imaging Sciences, Biomedical Imaging Institute, The University of Manchester, Manchester, United Kingdom; ^5^ Department of Empirical Inference, Max Planck Institute for Intelligent Systems, Tübingen, Germany

**Keywords:** information theory, causal strength, graphical models, transfer entropy, structural equations, neural oscillations

## Abstract

**Introduction:** Transient phenomena play a key role in coordinating brain activity at multiple scales, however their underlying mechanisms remain largely unknown. A key challenge for neural data science is thus to characterize the network interactions at play during these events.

**Methods:** Using the formalism of Structural Causal Models and their graphical representation, we investigate the theoretical and empirical properties of Information Theory based causal strength measures in the context of recurring spontaneous transient events.

**Results:** After showing the limitations of Transfer Entropy and Dynamic Causal Strength in this setting, we introduce a novel measure, relative Dynamic Causal Strength, and provide theoretical and empirical support for its benefits.

**Discussion:** These methods are applied to simulated and experimentally recorded neural time series and provide results in agreement with our current understanding of the underlying brain circuits.

## 1 Introduction

During both wakefulness and sleep, the mammalian brain is able to implement numerous functions key to our survival with extraordinary reliability. This implies precise coordination of transient *mechanisms* at multiple spatiotemporal scales ensuring both the synergy between brain regions contributing to the same task, and the non-interference between network activities in charge of different functions. Evidence for such transient mechanisms is provided by the variety of neural events that can be observed in brain activity across multiple structures. Such phenomena may occur in response to stimuli, as has been observed for gamma oscillations (Tallon-Baudry and Bertrand, 1999; Fries, 2015), and may play a role in the dynamic encoding of information. However, key phenomena can also occur spontaneously, as exemplified by the variety of events occurring during sleep. These include Sharp Wave-Ripples (SWR) complexes that occur in the hippocampus during the same sleep stages, and take the form of a slow deflection (the sharp wave, SW) superimposed with a fast short-lived oscillation (the ripple). SWRs have been extensively studied and a large set of evidence supports their key role in episodic memory consolidation and the recall of previous experiences (Lee and Wilson, 2002; Diba and Buzsaki, 2007; Ego-Stengel and Wilson, 2010).

In order to understand how these transient phenomena operate mechanistically, causality measures based on observed neural time series can be very helpful to quantify the underlying transient influences between brain structures. Several measures of causality have been proposed, starting in the econometrics literature with Granger causality (GC) (Granger, 1969), relying on vector auto-regressive models. This measure can be generalized to an information-theoretic quantity: Transfer Entropy (TE) (Schreiber, 2000). In the present work, we focus on “model-free” quantities such as TE that are defined independently of the specific functional relationships entailed by a particular model of the dynamics. TE and GC have been used to assess the significance of causal links, but also the “strength” of these links. However, whether they are appropriate quantities to measure such strength is debated (Janzing et al., 2013; Stokes and Purdon, 2017).

Structural Causal Models (SCM, see Supplementary Section SA for background) also allow causal strength measures to be evaluated by their ability to reflect the magnitude of the changes resulting from removing the causal links. In this context, the relevance of causality measures has been investigated by Ay and Polani (2008), who discuss how to account for the effect of knockout experiments, and introduce a measure of *information flow*, emphasizing its desirable properties; Janzing et al. (2013) provide interesting theoretical justifications for this kind of measure and extend it to define *causal strength* (CS) of an arbitrary set of arrows in a graphical model. With respect to TE, information flow and CS have the benefit to be local, in the sense that they depend only on the direct causes of the observed effects and their associated mechanisms. This makes CS a good candidate to measure transient connectivity changes during non-stationary neural events, as they would be able to restrict themselves to causal influences that take place at a specific time, associated to specific arrows in the “unrolled” causal graph describing time-varying interactions.

However, we will argue that, even in simple unidirectional settings where a “source” region is driving events in a target region, such TE and CS may not reflect well a key element for neuroscientists: the role played by transient dynamics. Based on the potential outcome framework (Rubin, 1974), causal reasoning has also been used to provide intuitive measures of the *causal impact* of a specific phenomenon happening at a given time point (Brodersen et al., 2015), by comparing it to a putative scenario where this phenomenon does not happen and called *synthetic control* in economics (Abadie, 2021). This inspired us to take into account the peri-event change of signals compared to a pre-event stage as another component of causal influence.

Therefore, we look at causal influences through the lens of interventions in SCMs to propose a principled quantification of the strength of causal interactions in peri-event time series, i.e., datasets collected specifically around the times of occurrence of an identified phenomenon in neural signals. Based on information theoretic analyses, we assess the relevance and issues raised by a time-varying implementation of GC, TE and causal strength (DCS, where D stands for *Dynamic*), and extend DCS to a novel measure, the relative DCS (rDCS), to quantify causal influences reflected by both the connectivity and the event-related change at the cause. We show theoretically that rDCS is effective in uncovering dynamic causal influences for task-dependent events that are often accompanied with a deterministic component, as well as for spontaneous events. We also demonstrate how choices made for aligning peri-event time series collected across multiple occurrences of these events may bias causality measures, and we propose a way to align the detected events favoring the recovery of the ground truth causal direction for a uni-directionally coupled system. The benefits of rDCS over TE and DCS is demonstrated by both simulated toy models and neurophysiological recordings of SWRs. Overall, our results suggest that rDCS helps better quantify the causal interactions between transient dynamical events, and thus uncover elementary mechanisms that shape brain activities.

## 2 Methods

### 2.1 General principles for the analysis of event-related causal interactions

#### 2.1.1 Structural Causal Models (SCM)

**FIGURE 1:**
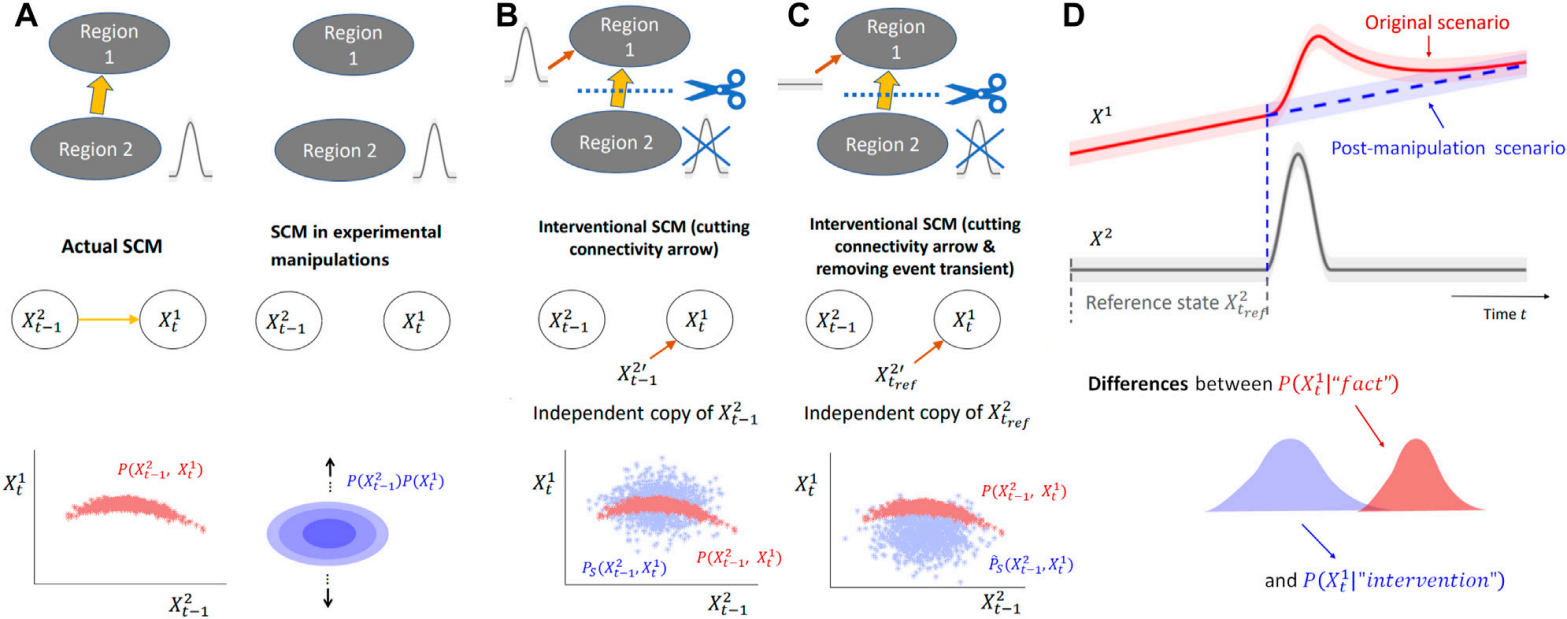
Analysis of event-related causality via interventions in SCMs. **(A)** (Top) Diagrams representing two brain regions with uni-directional connectivity from Region 2 to Region 1 and the post-manipulation scenario where the connectivity is removed. Region 2, as the “cause region”, exhibits transient events (grey) that influence Region 1 by propagating along the anatomical connection. (middle) SCMs underlying the diagrams, where $X_{t}^{1}$ and $X_{t}^{2}$ denotes states of Region 1 and Region 2. (bottom left) joint distribution of the two nodes in the SCM above reflecting dependencies between them. (bottom right) Hypothetical Gaussian joint distribution of two nodes in the SCM above with black arrows indicating varied covariance. **(B)** (Top) An experimental manipulation of the two-region diagram in **(A)** related to the intervention in measuring causal strength: cutting the anatomical connectivity. (middle) A corresponding intervention of the SCM in **(A)** represents cutting the causal arrow and feeding the effect node $X_{t}^{1}$ with an independent copy of the cause node $X_{t-1}^{2}$ . (bottom) joint distribution of the corresponding intervention distribution in contrast to the joint distribution in (A, bottom left). **(C)** (Top) Another experimental manipulation of the two-region diagram in **(A)**: cutting the anatomical connectivity and removing the event-based signal changes at Region 2. (Bottom) The corresponding intervention of the SCM in **(A)** represents cutting the causal arrow and feeding the effect node $X_{t}^{1}$ with an independent copy of a reference state of the cause node $X_{t_{\mathrm{ref}}}^{2}$ . (bottom) joint distribution of the corresponding intervention distribution in contrast to the joint distribution in (A, bottom left). **(D)** (Top) A time course of observed peri-event signals of Region 1 ( $X_{t}^{1}$ , red) and Region 2 ( $X_{t}^{2}$ , grey) reflecting the original scenario. The blue dashed time course represents the post-intervention scenario where $X_{t}^{1}$ evolves without the influence from $X_{t}^{2}$ . The interval marked by grey dashed lines refers to the reference state before the occurrence of events in $X_{t}^{2}$ . (Bottom) Visualization of the difference between the original and post-intervention scenarios at each time point.

**FIGURE 2:**
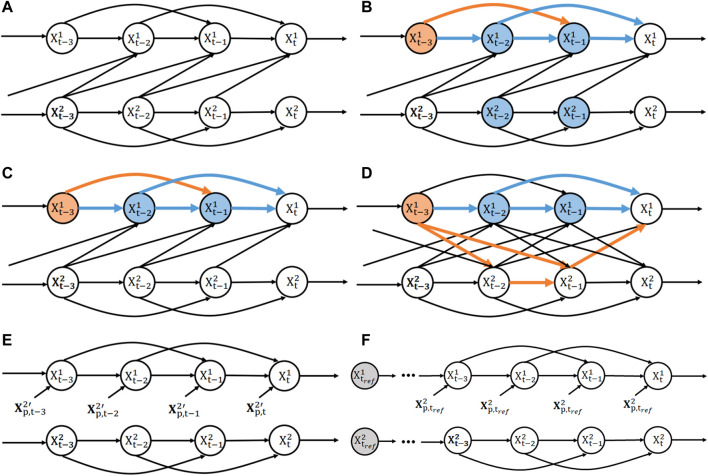
D-separation of bi-variate SVAR(2) model. **(A)** Structural causal model of a bi-variate SVAR(2) model defined in Eq. 2a with uni-directional coupling from *X* ^2^ to *X* ^1^. **(B)** Conditioning on both past states of *X* ^1^ and *X* ^2^ blocks all paths from $X_{t-3}^{1}$ to $X_{t}^{1}$ . Blue nodes represents conditioned nodes while blue arrows marks blocked paths. Orange arrows marks the unblocked paths. **(C)** Conditioning on past states of *X* ^1^ alone blocks all paths from $X_{t-3}^{1}$ to $X_{t}^{1}$ in the uni-directional case. Color codes are the same as **(B)**. **(D)** Conditioning on past states of *X* ^1^ alone does not block all paths from $X_{t-3}^{1}$ to $X_{t}^{1}$ in the bi-directional case. Color codes are the same as **(B)**. **(E)** The intervention implemented in devising CS is to break the causal arrows and send an independent copy $X_{p,t}^{2}$ to $X_{t}^{1}$ at each time point. This diagram applies to both CS and DCS (Section 2.2.3). **(F)** The intervention implemented in devising rDCS is to break the causal arrows and send an independent copy of the stationary state $X_{p,t_{\mathrm{ref}}}^{2}$ (marked by grey) to $X_{t}^{1}$ at each time point.

Mathematically, an SCM for variables {*V*_1_, …, *V*_*d*_} is a collection of so-called “structural assignments” of the form

$$V_{j}:=f_{j}\left({PA}_{j},N_{j}\right),\;j=1,\ldots ,d.$$ (1)

where the right hand side function *f*_*j*_ determines the assignment of the value of *V*_*j*_ on the left-hand side based on the values of parents variables **PA**_*j*_ ⊂ {*V*_1_, …, *V*_*d*_} and of the so-called exogenous random variable *N*_*j*_. The SCM is associated to a directed graph, the *causal graph*, where each variable *V*_*j*_ is represented by a node, and the parent-child relations between them is indicated by a “parent → child” arrow. While SCMs do not necessary include time information, we can exploit them to study dynamic interactions between two subsystems by considering causal relations linking variables representing one subsystem at past time points, to variables representing the other subsystem at a future time point. As an example, Figure 1A shows two uni-directionally coupled brain regions whose activities are represented by time series $\left\{\ldots ,X_{t}^{1},X_{t+1}^{1},\ldots \right\}$ and $\left\{\ldots ,X_{t}^{2},X_{t+1}^{2},\ldots \right\}$ and the corresponding SCM links the past of *X* ^2^ to the future of *X* ^1^. Typically, such a model also includes dependencies of each regional activity on its own past, and those dependencies can involve multiple time steps, leading to causal graphs of the form exemplified in Figure 2A. We focus on information theoretic causality measures, which are typically “model free”, in the sense that they can be expressed independently from the choice of functions *f*_*j*_ in the assignments of Eq. 1. However, model-free estimation of information theoretic quantities is a challenging problem that we will not address in this paper. Instead, estimation of the relevant quantities will rely on the following linear time-inhomogeneous Structural Vector Autoregressive (SVAR) model.

$$X_{t}^{1}:=a_{t}^{⊤}X_{p,t}^{1}+b_{t}^{⊤}X_{p,t}^{2}+\eta_{t}^{1},\;\eta_{t}^{1}∼N\left(k_{t}^{1},\;\sigma_{1,t}^{2}\right),$$ (2a)

$$X_{t}^{2}:=c_{t}^{⊤}X_{p,t}^{1}+d_{t}^{⊤}X_{p,t}^{2}+\eta_{t}^{2},\;\eta_{t}^{2}∼N\left(k_{t}^{2},\;\sigma_{2,t}^{2}\right).$$ (2b)

where:

• $\eta_{t}^{k}$ is the innovation for channel *k* at time *t*, sampled independently from the past values of *X* and from innovations at other time points and/or channels,
• $X_{p,t}^{k}={\left[X_{t-1}^{k},\ldots ,X_{t-p}^{k}\right]}^{⊤}$ is the vector collecting past *p* samples of the time series,
• the *t* subscript in all parameters $\left(a_{t},\;b_{t},\;c_{t},\;d_{t},\;k_{t}^{1},\;k_{t}^{2},\sigma_{1,t}^{2},\sigma_{2,t}^{2}\right)$ comes from our time-inhomogeneity assumptions and is not standard in the GC literature.

Note that the constraint of independence between exogenous variables $\eta_{t}^{k}$ associated to different channels entails the assumption of no contemporaneous effects, in contrast to, e.g., Moneta et al. (2011). Figure 2A exemplifies the associated causal graph for *p* = 2 and *c*_*t*_ = 0, such that *X* ^2^ is dependent only on *X*_2_ itself, encodes unidirectional causation from *X* ^2^ to *X* ^1^. The methods presented in this paper can be applied to time series generated by any other Markovian time series model (e.g., non-linear models (Marinazzo et al., 2011a)). However, the choice of SVAR 1) allows expressing the link between Granger causality and Transfer Entropy, 2) facilitates the estimation of all information theoretic quantities (which is inherently hard in a model-free setting, see, e.g., McAllester and Stratos (2020), as they get an analytic expression based on second order statistics), 3) avoids issues related to the non-parametric estimation of information theoretic quantities (e.g., finite sample bias), 4) allows to build easily interpretable models of transient oscillations.

#### 2.1.2 Interventions in SCMs

One key question in causality is estimating the effect of (possibly imaginary) *manipulations* (see, e.g., Woodward, 2001) of the system of interest from data, which boils down to comparing two “worlds” or scenarios (Shpitser and Pearl, 2008): the original world where no manipulation is performed, and the “post-manipulation” world.

Both original and post-manipulation worlds typically cannot be measured simultaneously (e.g., “treatment” and “no treatment” in the same patient). However, estimating their differences arguably forms the basis of causal investigations in empirical sciences, for example, by performing randomized experiments on multiple instances of a system designed with mutually exclusive treatments to infer the outcome of manipulations of this system. However, even performing carefully controlled experiments on close to identical instances of a system is often challenging in reality, as many physical and physiological phenomena cannot be easily reproduced or manipulated. This is typically the case for *spontaneous* transient neural events investigated in this paper, where neurophysiological experimental techniques limit the understanding and control of their conditions of occurrence, as well as the ability to precisely modify some aspects of network activity to test assumptions on the underlying mechanisms.

Under additional model assumptions, such as the absence of unobserved confounders, the framework of SCMs (as briefly introduced in Section 2.1.1 and Supplementary Section SA), can be leveraged to infer the outcome of manipulations based on observational data only. Assuming those assumptions are met (see also Discussion for examples), the SCM inferred from data can be modified using a family of operations named *interventions* to model the manipulations of the system described by the SCM (Pearl, 2000; Peters et al., 2017). Intervening typically refers to modifying the structural equation associated to one node in the SCM, to study the modifications it brings about in the system. When interventions are performed, the only affected mechanistic relations (represented by arrows in an SCM) are the ones between the intervened nodes and their parent nodes. For instance, one can impose a fixed deterministic value to a node, or that this node’s variable is drawn from a given distribution, independently from other variables in the SCM (Janzing et al., 2013; Correa and Bareinboim, 2020; Peters et al., 2017, Chapter 3). Both such interventions lead to an intervened causal graph where the arrows between the node intervened upon and its parents are removed. For a better understanding of the manipuation modelled in these two cases, consider examples from experimental manipulations in electrophysiology. The first case involves fixing a value, as in voltage clamp techniques used to study channel conductance. By fixing the membrane potential of a single neuron, the causal arrow between the membrane potential and extracellular ion concentration is broken. An example of the second case is injecting current in plasticity studies to maintain certain firing rates in a patch-clamped neuron, ensuring that the pharmacological shutdown of certain ion channels does not cause a change in neuronal firing patterns.

Importantly, while an intervention modifies the graph associated to an SCM, the variables’ joint distribution can still be obtained by exploiting interventional knowledge, (unintervened) observational data and prior assumptions related to the unaffected conditionals.

For the SCM introduced in Eq. 1, intervening on *V*_*k*_ consists in replacing its structural assignment by a new one:

$$V_{k}:=\tilde{f_{k}}\left(\tilde{{PA}_{k}},\tilde{N_{k}}\right).$$ (3)

where the new function $\tilde{f_{k}}$ , set of parents $\tilde{{PA}_{k}}$ , and/or the distribution of the exogenous variable $\tilde{N_{k}}$ may be differ from the original ones. The resulting distribution ${\tilde{P}}_{V}$ is called *intervention distribution* and denoted $P_{V}^{\text{ do}\left(V_{k}:=\tilde{f_{k}}\left(\tilde{{PA}_{k}},\tilde{N_{k}}\right)\right)}$ (see e.g., Peters et al. (2017, chapter 6)), where the superscript indicates that we refer to the distribution resulting from the modification of the SCM’s *k*th equation by Eq. 3, which is called a “do operation”. Meanwhile, the other structural equations and the distribution of their associated exogenous variables are kept unchanged.

#### 2.1.3 From causal strength to a measure of event-based causal influence

Typically, one brain region influences another through axonal propagation of spiking activity in afferent neurons, and synaptic transmission to dendrites of the target region. In the simplest bi-variate case (i.e., if we focus on two brain regions with direct synaptic projections), this relationship can be represented in an SCM by Figure 1A(left) where two nodes representing the neural activities of the two regions are linked by a uni-directional arrow. For the sake of simplicity, we ignore for now the influence of the past of *X* ^2^ on itself, such that the only causal link is from the past (at *t* − 1) of region 2 to the present (at *t*) of region 1. The joint probability can be causally factorized as $P\left(X_{t-1}^{2},X_{t}^{1}\right)=P\left(X_{t}^{1}|X_{t-1}^{2}\right)P\left(X_{t-1}^{2}\right)$ , where $P\left(X_{t}^{1}|X_{t-1}^{2}\right)$ reflects the stochastic map or causal mechanism between the child and parent of the arrow: for example, Figure 1A(bottom left) shows a typical example how a nonlinear structural equation (i.e., the arrow) induces dependencies between a normally-distributed $X_{t-1}^{2}$ and $X_{t}^{1}$ .

A natural manipulation to study the causal mechanism is to shut down the synaptic transmission during event occurrence and compare the outcomes, e.g., via physically cutting the pre-synaptic dendrite attached to the synapse, or pharmacological blockade of the relevant ion channel. Suppose that the experiment could be done, the data obtained in this hypothetical manipulated scenario can be modeled by another SCM without the arrow between $X_{t-1}^{2}$ and $X_{t}^{1}$ , as seen in Figure 1A(right middle), such that they are independent from each other with $\tilde{P}\left(X_{t-1}^{2},X_{t}^{1}\right)=\tilde{P}\left(X_{t}^{1}\right)\tilde{P}\left(X_{t-1}^{2}\right)$ due to the Markov properties (Supplementary Section SA). In a model-free setting and in absence of data where this (technically challenging) manipulation is actually done, the choice of factorized $\tilde{P}$ , such as the marginal mean and covariances, is non-trivial (see illustration in Figure 1A(bottom right)). Janzing et al. (2013) introduce a well-grounded way to make this choice to emulate this experimental ablation of connectivity, that generalize to arbitrary causal graphs.

This approach will be thoroughly discussed in Section 2.2.3 but here we explain it briefly in this simplified example to provide the readers with an intuitive understanding of the principles. Figure 1B illustrates the intervention performed in the SCM: cut the causal arrow from $X_{t-1}^{2}$ to $X_{t}^{1}$ and feed $X_{t}^{1}$ with an independent copy of $X_{t-1}^{2}$ (denoted $X_{t-1}^{2^{\prime }}$ ), where *independent copy* means that $X_{t-1}^{2^{\prime }}$ is a random variable statistically independent from any exogenous variables within the graph, and with the same marginal distribution as $X_{t-1}^{2}$ . The idea of this intervention is to achieve the independence of variables in the post-manipulation world by exploiting the observational conditional and marginal probabilities available to us. That is:

• choose $\tilde{P}\left(X_{t-1}^{2}\right)$ to be the observed marginal distribution of the cause, i.e., $\tilde{P}\left(X_{t-1}^{2}\right)=P\left(X_{t-1}^{2}\right)$ , because it is the only one available to us in a model-free setting. Consider alternative choices: we could set the cause to a constant, but which constant to choose is unclear without additional information on the system. For example, even taking the observational average would not be realistic if $X_{t-1}^{2}$ is binary,
• replace the cause → effect mechanism $P\left(X_{t}^{1}|X_{t-1}^{2}\right)$ by the operation of feeding the effect node $X_{t}^{1}$ with an independent copy of the cause node $X_{t-1}^{2}$ at the same time *t* − 1, such that the mechanism becomes $\overset{\sim }{P}\left(X_{t}^{1}\right)=\int P\left(X_{t}^{1}|X_{t-1}^{2^{\prime }}\right)P\left(X_{t-1}^{2^{\prime }}\right)dX_{t-1}^{2^{\prime }}=P\left(X_{t}^{1}\right)$ . Here again, the choice of observational density guarantees that the resulting mechanism is well defined for arbitrary SCMs.

The strength of the causal arrow is then quantified by the Kullback-Leibler (KL) divergence *D*_*KL*_ between original (unintervened) and intervened joint densities $D_{KL}\left(P\|\tilde{P}\right)=D_{KL}\left(P\left(X_{t-1}^{2},X_{t}^{1}\right)\|P\left(X_{t-1}^{2}\right)P\left(X_{t}^{1}\right)\right),$ which in this simple case thus boils down to the mutual information between the two nodes. Figure 1B(bottom) illustrates the contrast between the actual joint distribution and the intervention distribution for such intervention: by comparing these two distributions one could quantify how much the causal mechanism tilts the Gaussian shape, while they still largely overlap. In a context where nodes correspond to single neurons, this can be thought of as a proxy for the experiment of cutting the axon of afferent neurons, while injecting a current to maintain the baseline level of excitation in the target neuron, such that it is kept in naturalistic conditions.

However, we will argue that this choice of intervention is not an ideal way to measure the event-based causal influences between two brain regions. Going back to the manipulation experiment, despite the transient activities occurring at the pre-synaptic neurons as an input to the synapse, due to the cutoff of afferents or blockage of ion channels, the activity of the post-synaptic neuron (i.e., the effect variable) is expected to remain at baseline level without being influenced by the event occurring in the cause. In this context, the operation of feeding the effect node $X_{t}^{1}$ with an independent copy of the cause node $X_{t-1}^{2}$ at the same time *t* − 1 still implicitly incorporates the influence of the event-related transient changes undergone by *X* ^2^ at the time *t* − 1 on $X_{t}^{1}$ , as the distribution of $X_{t-1}^{2}$ may strongly differ from what it is during baseline activity (without the occurrence of events). Therefore we propose instead to reconstruct the baseline state by replacing the independent copy of the marginal distribution of the event-related activity $X_{t-1}^{2}$ by the marginal distribution of a baseline state in $X_{t_{\mathrm{ref}}}^{2}$ , at a reference time point *t*_*ref*_ where the event of interest has not yet occurred (Figure 1D(Top)), such that the new intervention distribution becomes $\tilde{P}\left(X_{t-1}^{2},X_{t}^{1}\right)=P\left(X_{t-1}^{2}\right)\int P\left(X_{t}^{1}|X_{t_{\mathrm{ref}}}^{2^{\prime }}\right)P\left(X_{t_{\mathrm{ref}}}^{2^{\prime }}\right)dX_{t_{\mathrm{ref}}}^{2^{\prime }}=P\left(X_{t-1}^{2}\right)\tilde{P}\left(X_{t}^{1}\right)$ . The difference between feeding independent copies of different marginal distributions and the resulting baseline joint probability are illustrated in Figures 1B, C. We thus argue that this is a better reference scenario for testing the influence of an event between two brain regions because it accounts for the event-related variations of the input distribution relative to baseline activity.

### 2.2 Candidate time-varying causality measures

We now present the time-varying versions of commonly-adopted causal strength measures of a given direction of causation *X* ^2^ → *X* ^1^ and discuss their properties in the context of transient event-based causality analysis, in light of the above principles. The candidate measures include time-varying extensions of Granger causality (GC), Transfer Entropy (TE) and Causal Strength (CS) (Janzing et al., 2013). To make the comparison quantitative, time series are modeled using the bivariate linear SVAR model of Eq. 2a and Eq. 2b. As we will see, all measures boil down to comparing the “full” bivariate model to a model where the contribution of the cause time series to the effect is removed in some way. Generalization to more than two observed time series is possible in all cases and briefly mentioned for each approach.

#### 2.2.1 Granger causality

Granger causality (GC), as well as its information-theoretic extension, Transfer Entropy (TE) is rooted in Wiener’s principle of causality. For the bivariate case, Granger (1969) defines the statement of (Granger-)causality from *X* ^2^ to *X* ^1^ whenever knowledge of $X_{p,t}^{2}$ , in addition to $X_{p,t}^{1}$ , yields a strictly better prediction of $X_{t}^{1}$ . This can be interpreted as a comparison between two prediction scenarios:

• Scenario 1: predict $X_{t}^{1}$ using both $X_{p,t}^{1}$ and $X_{p,t}^{2}$ ,
• Scenario 2: predict $X_{t}^{1}$ using only $X_{p,t}^{1}$ ,

where $X_{p,t}^{1}$ and $X_{p,t}^{2}$ refer to the respective *p* previous past points of each time series, without further specification, such that in our notation *p* can be potentially infinite.

The predictive model describing the first scenario is referred to as the *full model* (Geweke, 1984) and corresponds to Eq. 2a of the SVAR model, where the first variable *X* ^1^ is dependent on both variables *X* ^1^ and *X* ^2^. An estimate of the innovation variance of $X_{t}^{1}$ ( $\sigma_{1,t}^{2}$ in Eq. 2a) is the mean squared residual error $\left({\hat{\sigma }}_{1,t}^{2}\right)$ of the forecast of $X_{t}^{2}$ under the assumption that both $X_{p,t}^{1}$ and $X_{p,t}^{2}$ contribute to $X_{t}^{1}$ . Under Scenario 2 where $X_{t}^{1}$ is predicted only by $X_{p,t}^{1}$ , we have a *reduced model*

$$X_{t}^{1}=a_{t}^{\prime ⊤}X_{p^{\prime },t}^{1}+{\eta_{t}^{1}}^{\prime },\;{\eta_{t}^{1}}^{\prime }∼N\left({k^{\prime }}^{1},\;{\sigma^{\prime }}_{1,t}^{2}\right).$$ (4)

where the model order *p*′, the coefficient **a**′, the innovations mean ${k^{\prime }}^{1}$ and innovations variance ${\sigma^{\prime }}_{1}^{2}$ are different from the corresponding terms in Eq. 2a and are classically re-estimated.

If *X* ^2^ Granger-causes *X* ^1^, then the full model should fit the data more accurately compared to the reduced model as measured by the estimated variance ${\hat{\sigma^{\prime }}}_{1,t}^{2}$ , which should be larger than ${\hat{\sigma }}_{1,t}^{2}$ . Then the amount of Granger causality can be defined as the log ratio of the residual variance between the reduced model and the full model, which leads to estimating the magnitude of Granger causality as

$$\text{GC}\left(X_{t}^{2}\to X_{t}^{1}\right)=\frac{1}{2}\log\left(\frac{{{\hat{\sigma^{\prime }}}_{1,t}}^{2}}{{{\hat{\sigma }}_{1,t}}^{2}}\right),$$ (5)

where the factor 1/2 is chosen for consistency with TE (see Section 2.2.2). While the above linear SVAR model is the most widely used, Granger causality has been extended to non-linear models following the same predictive approach (e.g., Marinazzo et al., 2008; Marinazzo et al., 2011b; Diks and Wolski, 2016; Wismüller et al., 2021, for a recent review see Shojaie and Fox, 2022).

#### 2.2.2 Transfer Entropy

TE is an information-theoretic implementation of Wiener’s principle, where a comparison between the prediction performance of the above two scenarios is quantified with conditional entropy. TE quantifies to which amount *X* ^2^ causes *X* ^1^ in the Granger sense and is defined by the entropy difference

$$\text{TE}\left(X_{t}^{2}\to X_{t}^{1}\right)=H\left(X_{t}^{1}|X_{p,t}^{1}\right)-H\left(X_{t}^{1}|X_{p,t}^{1},X_{p,t}^{2}\right).$$ (6)

Interestingly, using the Kullback-Leibler (KL) divergence *D*_*KL*_ between two probability densities $D_{KL}\left(p\|q\right)=\int p\left(x\right)\log\frac{p\left(x\right)}{q\left(x\right)}dx,$ TE can be rewritten as an expected KL-divergence between the corresponding conditional probabilities, thereby contrasting the two above mentioned scenarios:

$$\text{TE}\left(X_{t}^{2}\to X_{t}^{1}\right)=E_{X_{p,t}^{1},X_{p,t}^{2}}\left[D_{KL}\left(p\left(X_{t}^{1}|X_{p,t}^{1},X_{p,t}^{2}\right)\|p\left(X_{t}^{1}|X_{p,t}^{1}\right)\right)\right].$$ (7)

As noticed by Barnett et al. (2009), under stationary Gaussian SVAR assumptions the analytic expression of Gaussian entropy applied to Eq. 6 leads to $\text{GC}\left(X_{t}^{2}\to X_{t}^{1}\right)=\text{TE}\left(X_{t}^{2}\to X_{t}^{1}\right)$ in the limit of unbiased variance estimation, such that TE appears as a strict generalization of GC, and can be estimated by GC in the context of Gaussian SVAR models. TE and GC statistics are two commonly used measures of causal strength for investigating interactions between brain regions (e.g., Vicente et al., 2011; Besserve et al., 2010, 2015; Ding et al., 2006; Wibral et al., 2013, 2014; Barrett et al., 2010; Wen et al., 2013; Shorten et al., 2021; Cekic et al., 2018, with several widely applied toolboxes such as Barnett and Seth, 2014; Montalto et al., 2014; Lizier, 2014; Wollstadt et al., 2019). They generalize easily to more than two signals by including also the past of additional signals in the prediction equations when assessing causality for a specific pair, as is done in conditional pairwise Granger causality (Barrett et al., 2010; Faes et al., 2011; Runge et al., 2012; Barnett and Seth, 2014). Based on the observational conditional distribution of the neural signals being analyzed, these two measures estimate a quantity that is easily interpretable from a forecasting perspective. However, they have some limitations with regard to their interpretability as interventions in the SCM framework and in the time varying setting that interests us in the present paper.

A key issue is that the reduced model ignores but *does not remove* the influence of past values of ***X*** ^2^ $\left(X_{p,t}^{2}\right)$ on $X_{t}^{1}$ by marginalizing with respect to them. It can be shown that such change does not preserve the SCM structure, and leads to violations of the Markov properties due to the implicit dependency on the mechanisms relating $X_{p,t}^{2}$ and $X_{p,t}^{1}$ , which manifest themselves through the $p\left(X_{p,t}^{2}|X_{p,t}^{1}\right)$ term of the marginalization equation (Ay and Polani, 2008; Janzing et al., 2013):

$$p\left(X_{t}^{1}|X_{p,t}^{1}\right)=\int p\left(X_{t}^{1}|X_{p,t}^{1},X_{p,t}^{2}\right)p\left(X_{p,t}^{2}|X_{p,t}^{1}\right)dX_{p,t}^{2}.$$ (8)

As a consequence, the reduced model cannot be generally interpreted as an intervention on the original SCM that would result in a model where arrows associated to the causal influence of interest would be removed. In addition, in case of bi-directional coupling, the reduced model of Eq. 4 is misspecified (in a generic case) for any finite order. This can be seen easily by exploiting the *d*-separation criterion (Supplementary Section SA), as illustrated in Figure 2. Figure 2B shows the estimation in the full model, where conditioning on both $X_{p,t}^{1}$ and $X_{p,t}^{2}$ blocks all the paths from $X_{t-3}^{1}$ to $X_{t}^{1}$ such that $X_{t-3}^{1}$ and $X_{t}^{1}$ are conditionally independent. For such a uni-directionally-coupled system, a finite order for the reduced model also guarantees such conditional independence, as seen in Figure 2C where all paths are blocked by conditioning. However, in the same system with bi-directional coupling, for any *k* > *p* (i.e., *k* > 2), there is always a path from $X_{t-k}^{1}$ to $X_{t}^{1}$ going through nodes of *X* ^2^ that is unblocked by $\left(X_{t-p}^{1},\;\ldots ,X_{t-1}^{1}\right)$ . As Figure 2D shows, 2 paths from $X_{t-3}^{1}$ to $X_{t}^{1}$ are not blocked by conditioning on $X_{p,t}^{1}$ . Under faithfulness assumptions, this implies that there is conditional dependence between $X_{t}^{1}$ and its remote past samples, no matter how many finite past states we are conditioning on. This further implies that to minimize the forecast error of $X_{t}^{1}$ in the reduced model one should ideally exploit the past information of this time series up to *p* = +*∞*.

This issue has been both raised and addressed in the literature, in particular by resorting to Autoregressive Moving Average models and state space models for defining an appropriate reduced model (e.g., (Barnett and Seth, 2015; Solo, 2016)). However, this remains an important limitation when extending TE to time-varying versions, where the model is assumed to be stationary at best locally in time. For example, when defining a non-stationary SVAR model as Eq. 2a, we assume a different linear model in each 1-point time window. The non-locality of TE is particularly problematic for such a time-varying model assumption because of the implicit influence of past activities on this quantity.

#### 2.2.3 Dynamic causal strength

To overcome the limitations of TE and GC, Ay and Polani (2008) have proposed a measure of *information flow* to quantify the influence of some variables on others in a system, which has been further studied and generalized in Janzing et al. (2013) as a measure of the *Causal Strength* (CS) of an arbitrary set of arrows in a graphical model. In the present paper, we define CS in the context of time-inhomogeneous vector autoregressive processes and their associated unrolled causal graph, and thus call it *Dynamic Causal Strength* (DCS).

DCS can be naturally defined using the SCM interventional formalism (Pearl (2000); Peters et al. (2017), see Section 2.1.2). Briefly, interventions are performed on nodes in order to remove the specific arrows from the causal graph whose influence we wish to quantify. In agreement with Ay and Polani (2008) and Janzing et al. (2013), in the context of inhomogeneous SVAR models (as illustrated in Figure 2A), an appropriate intervention to remove the causal influence from $X_{p,t}^{2}$ to $X_{t}^{1}$ can be designed as the following intervention (shown in Figure 2E): *remove the arrow* $X_{p,t}^{2}\to X_{t}^{1}$ *by injecting instead* ${X_{p,t}^{2}}^{\prime }$ *, an independent copy of* $X_{p,t}^{2}$ *with the same joint distribution, in the original mechanism* $P\left(X_{t}^{1}|X_{p,t}^{1},X_{p,t}^{2}\right)$ *.* The intervention distribution *p* ^*DCS*^ models the post-interventional world after removing the causal arrow from $X_{p,t}^{2}$ to $X_{t}^{1}$ and results in the entailed conditional probability

$$\begin{array}{ll} p^{DCS}\left(X_{t}^{1}|X_{p,t}^{1}\right) & =p^{do\left(X_{t}^{1}:=f\left(X_{p,t}^{1},{X_{p,t}^{2}}^{\prime },\eta_{t}^{1}\right)\right)}\left(X_{t}^{1}|X_{p,t}^{1},X_{p,t}^{2}\right)\\ & =\int p\left(X_{t}^{1}|X_{p,t}^{1},X_{p,t}^{2}\right)p\left(X_{p,t}^{2}\right)dX_{p,t}^{2}, \end{array}$$

which does not depend on $p\left(X_{p,t}^{2}|X_{p,t}^{1}\right)$ anymore, in comparison to Eq. 8. DCS then quantifies the KL divergence between the distributions of $X_{t}^{1}|\left(X_{p,t}^{1},X_{p,t}^{2}\right)$ obtained in both worlds, such that

$$\text{DCS}\left(X_{t}^{2}\to X_{t}^{1}\right)=E_{X_{p,t}^{1},X_{p,t}^{2}}\left[D_{KL}\left(p\left(X_{t}^{1}|X_{p,t}^{1},X_{p,t}^{2}\right)|p^{\mathrm{DCS}}\left(X_{t}^{1}|X_{p,t}^{1}\right)\right)\right].$$ (9)

A parametric formulation under linear Gaussian model assumptions is given in Supplementary Section SD.5. Generalization to more than two time series is also straightforward following (Janzing et al., 2013): *p* ^*DCS*^ and DCS are simply computed by also including conditioning on the past of all other time series, in addition to $X_{p,t}^{1}$ .

**Remark:** In contrast with Janzing et al. (2013), but in line with Ay and Polani (2008), we do not use jointly independent copies of each component of $X_{p,t}^{2}$ , that is, the copy preserves the dependency between the successive past time points of *X* ^2^. Indeed, Janzing et al. (2013) require having copies with jointly independent components in order to assess the individual strength of each arrow in the causal graph, which would correspond to the influence of each time lag in our setting. In contrast, this is not a requirement for us as we are only interested in assessing the overall effect of the whole past of a given time series on the another. One benefit of our choice is that it is consistent with the definition of TE: one can easily check that in absence of dependency of $X_{t}^{1}$ on its own past, both TE (based on Eq. 6) and DCS reach the same value: the mutual information of $X_{p,t}^{2}$ and $X_{t}^{1}$ . Given that successive samples may be strongly correlated in practice, our choice avoids unnecessary discrepancies between these two measures to focus on their key difference. In additional, our choice can be seen as in line with Janzing et al. (2013) when considering a state representation of the time series’ causal graph, where the node of variable *k* at time *t* would be the vector ${\left[X_{t}^{k},X_{t-1}^{k},\ldots ,X_{t-p+1}^{k}\right]}^{⊤}$ .

### 2.3 Near deterministic behavior of TE and DCS

The analysis of transient neural events leads us to analyze signals that have limited stochasticity in several respects: on the one hand, strongly synchronized oscillatory signals can be represented by SVAR models with low innovation variance, relative to the variance of the measured signal. Moreover, when a study focuses on a reproducible type of transient pattern, it is often characterized by a waveform that has little variability across collected trials. Such a situation can be modeled with a time-varying deterministic innovation, exhibiting strong variation of its mean across time, but no or little variance. We investigate the theoretical properties of TE and DCS in this regime, showing a benefit of DCS with respect to TE, but also remaining limitations.

#### 2.3.1 TE behavior for strongly synchronized signals

Besides, it has also been pointed out that the definition of TE in Eq. 7 has some other non-intuitive implications (Ay and Polani, 2008; Janzing et al., 2013). In particular, there are situations in which TE(*X* ^2^ → *X* ^1^) almost vanishes, although the influence is intuitively clear. How frequent are the practical situations in which we have these detrimental effects is unclear; however, theoretical analysis suggests that this can happen when the time series are strongly correlated.

To see this, we can derive from Eq. 7 the case where *X* ^2^ is a deterministic function of *X* ^1^ such that TE vanishes. Take the special case where $X_{t}^{2}$ is proportional to $X_{t}^{1}$ such that $X_{t}^{2}=kX_{t}^{1}$ , representing a time-wise synchronization of the two signals, the conditional variance will be

$$\begin{array}{ll} \Sigma_{X_{p}^{2}|X_{p}^{1}} & =\Sigma_{X_{p}^{2}}-\Sigma_{X_{p}^{2}X_{p}^{1}}\Sigma_{X_{p}^{1}}^{-1}\Sigma_{X_{p}^{1}X_{p}^{2}}\\ & =\Sigma_{X_{p}^{2}}-k\Sigma_{X_{p}^{2}}\cdot \left(\frac{1}{k^{2}}\Sigma_{X_{p}^{2}}^{-1}\right)\cdot k\Sigma_{X_{p}^{2}}=0 \end{array}$$

Plugging into Supplementary Equation S8 in Supplementary Section SD.4 yields,

$$\begin{array}{ll} \text{TE}\left(X_{t}^{2}\to X_{t}^{1}\right) & =\log\frac{b_{t}^{⊤}\text{Cov}\left[X_{p,t}^{2}|X_{p,t}^{1}\right]b_{t}+{\sigma_{1,t}}^{2}}{{\sigma_{1,t}}^{2}}\\ & =\log\frac{{\sigma_{1,t}}^{2}}{{\sigma_{1,t}}^{2}}=\log1=0 \end{array}$$

However, a strong correlation between two observed time series does not necessarily imply that causal interactions between them are weak, from an SCM perspective. We will investigate this case in Section 3.1 and compare with the results of DCS to show that DCS does not suffer from this non-intuitive vanishing problem.

#### 2.3.2 Insensitivity of TE and DCS to deterministic perturbations

While several intuitive properties make DCS a good candidate to quantify causal influences, we exhibit a counterintuitive property common to TE and DCS in the context of peri-event time series. Transient neural events are mainly investigated in two types of analyses: 1) stimulus-triggered (or response-triggered) data that are temporally aligned by task (or response) onset and 2) event-triggered data where occurrences of a type of brain-activity pattern are detected along the time course of the recordings (manually or algorithmically) and used to create peri-event trials.

In both cases, neural activities are likely to have a deterministic component appearing in the peri-event ensembles, due the similarity of the response to successive stimuli in case 1), or due to the similarity of the neural patterns detected in the recordings in case 2). Here we will show that, in a linear setting, TE and DCS are insensitive to such a deterministic component. Specifically, TE and DCS values are unaffected by interventions on the innovations’ mean at any time point.

First, we exhibit the role played by a deterministic perturbation in an example.

**Example 1**. *Consider the bi-variate SVAR(1) model in the following form*

$$X_{t}^{1}:=aX_{t-1}^{1}+bX_{t-1}^{2}+\eta_{t}^{1},$$ (10a)

$$X_{t}^{2}:=\eta_{t}^{2},$$ (10b)

*with* *a*, *b* ≠ 0 *and a stationary innovation for* *X* ^1^ *,* $\eta_{t}^{1}∼N\left(0,1\right)$ *, but a non-stationary innovation for* *X* ^2^ *,* $\eta_{t}^{2}∼N\left(\alpha \delta_{t,\;t_{0}},\sigma_{2,t}^{2}\right)$ *, with*

$$\delta_{t,t_{0}}=\left\{\begin{array}{cc} 1,\; & \mathrm{for}\;t=t_{0},\\ 0,\; & \mathrm{otherwise}. \end{array}\right.$$

When varying *α*, this models a intervention on the second time series. Then it can be easily shown that the expected time course of *X* ^1^ is

$$E\left[X_{t}^{1}\right]=\left\{\begin{array}{cc} \alpha ba^{t-t_{0}+1},\; & t\geq t_{0}+1\\ 0,\; & \text{otherwise.} \end{array}\right.$$

This witnesses the causal influence of $X_{t_{0}}^{2}$ on values of $X_{t}^{1}$ at subsequent times, which for large *α* results in large deviations from the baseline expectation of $X_{t}^{1}$ for *t* prior to *t*_0_. Intuitively, one may expect that a quantification of the magnitude (strength) of the causal influence of *X* ^2^ on *X* ^1^ should be larger for larger *α*, as a transient of larger magnitude propagates from *X* ^2^ to *X* ^1^. From a neuroscientific perspective, this could model an experimental setting where one brain region is electrically stimulated with increasing strength to detect whether it is anatomically connected to another. Obviously, the magnitude of the stimulation is expected to be critical to elicit a response in the target region. However, TE and DCS actually turn out to be insensitive to such stimulation.

We will show this in the more general setting of the SVAR(*p*) model of Eq. 2a and Eq. 2b.

**Proposition 1**. *For linear SVAR models defined by Eq. 2a and Eq. 2b, TE and DCS measures are invariant to deterministic perturbations, i.e., to changes in the mean of the innovation’s distributions* $\left(k_{t}^{1},k_{t}^{2}\right)$ .

*Proof.* Without loss of generality, we will show invariance to an elementary intervention at a single time *t*_0_ that transforms $\eta_{t_{0}}^{2}$ to $\eta_{t_{0}}^{2}+\alpha$ , which boils down to changing the mean parameters of the innovation $k_{t_{0}}^{2}$ in Eq. 2a and Eq. 2b. By linearity and symmetry of the problem for channel 1 and 2, invariance to deterministic perturbations results from combining several elementary interventions.

To compute how the intervention distribution of the new variables denoted $\left({\tilde{X}}^{1},{\tilde{X}}^{2}\right)$ changes with respect to the distribution of the original variables, we can examine the difference with respect to (*X* ^1^, *X* ^2^) that has the same innovations, except for $\eta_{t_{0}}^{2}$ for which we remove a constant *α*. (*X* ^1^, *X* ^2^) is then distributed according to the original distribution (before intervention), and the difference $\left(U,V\right)=\left({\tilde{X}}^{1}-X^{1},{\tilde{X}}^{2}-X^{2}\right)$ follows the equations

$$\begin{array}{ll} U_{t} & =a^{⊤}U_{p,t}+b^{⊤}V_{p,t},\\ V_{t} & =c^{⊤}U_{p,t}+d^{⊤}V_{p,t}+\delta_{t,\;t_{0}}, \end{array}$$

which is a set of linear deterministic difference equations with a unique solution making ***X*** and $\tilde{X}$ coincide before the intervention^1^ (*U*_*t*_, *V*_*t*_). As a consequence, by linearity, the interventional density $\tilde{p}$ is a shifted version of the original:

$$\tilde{p}\left(X_{t}^{1},X_{p,t}^{1},X_{p,t}^{2}\right)=p\left(X_{t}^{1}-U_{t},X_{p,t}^{1}-U_{p,t},X_{p,t}^{2}-V_{p,t}\right),$$

which implies the same for conditional marginal densities, e.g.,

$$\tilde{p}\left(X_{t}^{1}|X_{p,t}^{1},X_{p,t}^{2}\right)=p\left(X_{t}^{1}-U_{t}|X_{p,t}^{1}-U_{p,t},X_{p,t}^{2}-V_{p,t}\right)$$

and

$$\tilde{p}\left(X_{t}^{1}|X_{p,t}^{1}\right)=p\left(X_{t}^{1}-U_{t}|X_{p,t}^{1}-U_{p,t}\right).$$

As a consequence TE on the intervention distribution writes

$$\begin{array}{ll} \text{TE}\left({\tilde{X}}_{t}^{2}\to {\tilde{X}}_{t}^{1}\right) & =\int \tilde{p}\left(X_{t}^{1},X_{p,t}^{1},X_{p,t}^{2}\right)\log\frac{\tilde{p}\left(X_{t}^{1}|X_{p,t}^{1},X_{p,t}^{2}\right)}{\tilde{p}\left(X_{t}^{1}|X_{p,t}^{1}\right)}\;dX_{t}^{1}dX_{p,t}^{1}dX_{p,t}^{2}\\ & =\int p\left(X_{t}^{1}-U_{t},X_{p,t}^{1}-U_{p,t},X_{p,t}^{2}-V_{p,t}\right)\times \log\frac{p\left(X_{t}^{1}-U_{t}|X_{p,t}^{1}-U_{p,t},X_{p,t}^{2}-V_{p,t}\right)}{p\left(X_{t}^{1}-U_{t}|X_{p,t}^{1}-U_{p,t}\right)}dX_{t}^{1}dX_{p,t}^{1}dX_{p,t}^{2}. \end{array}$$

And by change of variable we get the invariance property:

$$\begin{array}{ll} \text{TE}\left({\tilde{X}}_{t}^{2}\to {\tilde{X}}_{t}^{1}\right)f & =\int p\left(X_{t}^{1},X_{p,t}^{1},X_{p,t}^{2}\right)\log\frac{p\left(X_{t}^{1}|X_{p,t}^{1},X_{p,t}^{2}\right)}{p\left(X_{t}^{1}|X_{p,t}^{1}\right)}\;dX_{t}^{1}dX_{p,t}^{1}dX_{p,t}^{2}\\ & =\text{TE}\left(X_{t}^{2}\to X_{t}^{1}\right), \end{array}$$

which can be generalized to arbitrary deterministic perturbations. The same reasoning can be applied to DCS leading to invariance as well (Supplementary Section SB) and this concludes the proof.

Arguably, this result is not what we would expect from an event-related measure of influence, because in the above example of Eq. 10a and Eq. 10b, setting a large *α* intuitively leads to a large influence of *X* ^2^ on *X* ^1^ provided *b* ≠ 0. Provided that TE and DCS can be made arbitrarily small by reducing the innovation’s variance $\sigma_{2,t}^{2}$ (according to their analytical expression in Supplementary Section SD), TE and DCS may detect no influence despite this strong effect on the mean of $X_{t}^{2}$ . Although this invariance result is rigorously derived for linear SVAR models, it uncovers an issue for non-linear models as well, the magnitude of the causal influence associated to deterministic perturbation then depending chiefly on the non-linear properties of the system under study, and not on the magnitude of the changes triggered by the perturbation. Moreover, linear SVAR(*p*) models being able to approximate nonlinear dynamics, this suggests that deterministic causal influences cannot be detected by TE or DCS for a broad class of models in practice.

As elaborated above, this is in contrast to what would be expected in the neuroscientific context, and directly relates to the observational, event-related setting that we investigate: the deterministic component is due to the alignment of the data with respect to an event of interest, and we do not have a different condition to contrast the occurrence of this event with what would have happened in its absence. This analysis calls for building a synthetic baseline condition that would allow deterministic changes to be detected.

### 2.4 A novel measure: relative Dynamic Causal Strength

#### 2.4.1 Motivation

Following the guidelines for event-based causality (presented in Section 2.1), we propose a novel measure, the relative Dynamic Causal Strength (rDCS), as a modification of DCS. This measure aims at taking into account the influence of event-based changes in the cause signals independent from the connectivity (the mechanism), and notably those driven by deterministic exogenous inputs. In the specific problem we are investigating, the cause is the past states of *X* ^2^, denoted $X_{p,t}^{2}$ , while the mechanism can be represented by the model in Eq. 2a and symbolized by the corresponding causal arrow in the causal graph. DCS only deletes the causal arrow in the post-intervention scenario but preserves the event-related change in the cause itself.

In the case where *X* ^2^ is driven by a deterministic exogenous input in a transient window, the cause exhibits significant changes relative to baseline; thus, intuitively, the causal effect should also be enhanced even if the causal arrow remains the same (i.e., the coefficient **b** stays unchanged). Apart from intervening on the causal arrow, further intervention can be implemented on the cause node to construct a post-intervention scenario where the cause receives no time-varying innovations. Therefore, inspired by causal impact (Section 2.1.3) which characterizes the difference between the current state and a baseline state, we propose (additionally to DCS) to replace the marginal of $X_{p,t}^{2}$ by the marginal of $X_{p,t_{\mathrm{ref}}}^{2}$ , that we denote $p_{\mathrm{ref}}\left(X_{p,t_{\mathrm{ref}}}^{2}\right)$ , for a reference time *t*_*ref*_. The reference time *t*_*ref*_ is typically chosen to be a stationary period before the occurrence of the transient deterministic perturbations and statistics of $X_{p,t_{\mathrm{ref}}}^{2}$ can be averaged by statistics of $X_{p,t}^{2}$ within this period. This leads to the *relative Dynamic Causal Strength* (rDCS)

$$\text{rDCS}\left(X_{t}^{2}\;\to \;X_{t}^{1}\right)=E_{X_{p,t}^{1},X_{p,t}^{2}}\left[D_{KL}\left(p\left(X_{t}^{1}|X_{p,t}^{2},X_{p,t}^{2}\right)\left\|p^{do\left(X_{t}^{1}:=f\left(X_{p,t}^{1},X_{p,t_{\mathrm{ref}}}^{2},\eta_{t}^{1}\right)\right)}\left(X_{t}^{1}|X_{p,t}^{1},X_{p,t}^{2}\right)\right.\right)\right]$$ (11)

with

$$p^{do\left(X_{t}^{1}:=f\left(X_{p,t}^{1},X_{p,t_{\mathrm{ref}}}^{2},\eta_{t}^{1}\right)\right)}\left(X_{t}^{1}|X_{p,t}^{1},X_{p,t}^{2}\right)=\int p\left(X_{t}^{1}|X_{p,t}^{1},X_{p,t}^{2}\right)p_{\mathrm{ref}}\left(X_{p,t}^{2}\right)dX_{p,t}^{2}$$ (12)

The implementation of rDCS given a SVAR model is derived in Supplementary Section SD.6. Generalization to more than two time series can be done in the same way as for DCS, by including extra conditioning on the past of other time series for all quantities.

Intuitively, the term *relative* originates from the comparison between the current past state $X_{p,t}^{2}$ and the reference past state $X_{p,t_{\mathrm{ref}}}^{2}$ . It is then natural to predict that in the uni-directional case, rDCS(*X* ^2^ → *X* ^1^) = DCS(*X* ^2^ → *X* ^1^) for any reference time *t*_*ref*_ if *X* ^2^ is stationary because stationarity implies that the marginal distributions of $X_{p,t_{\mathrm{ref}}}^{2}$ and $X_{p,t}^{2}$ are identical. As a particular case, this result implies that a transient loss of causal link from *X* ^2^ to *X* ^1^ will lead to rDCS = 0, while for a stationary bivariate system, DCS = rDCS is constant.

#### 2.4.2 Sensitivity of rDCS to deterministic perturbations

The definition of rDCS implies sensitivity to deterministic perturbations. Indeed, taking the example in Section 2.3.2, the reference state $X_{p,t_{\mathrm{ref}}}^{2}$ is unaffected by the deterministic perturbation. Consequently, the translational invariance does not hold for the intervention distribution because

$$\begin{array}{ll} \text{rDCS}\left({\tilde{X}}_{t}^{2}\to {\tilde{X}}_{t}^{1}\right) & =\int \tilde{p}\left(X_{t}^{1},X_{p,t}^{1},X_{p,t}^{2}\right)\log\frac{\tilde{p}\left(X_{t}^{1}|X_{p,t}^{1},X_{p,t}^{2}\right)}{\int \tilde{p}\left(X_{t}^{1}|X_{p,t}^{1},X_{p,t}^{2}\right){\tilde{p}}_{\mathrm{ref}}\left(X_{p,t}^{2}\right)dX_{p,t}^{2}}\\ & dX_{t}^{1}dX_{p,t}^{1}dX_{p,t}^{2}=\int p\left(X_{t}^{1}-U_{t},X_{p,t}^{1}-U_{p,t},X_{p,t}^{2}-V_{p,t}\right)\log\frac{p\left(X_{t}^{1}-U_{t}|X_{p,t}^{1}-U_{p,t},X_{p,t}^{2}-V_{p,t}\right)}{\int p\left(X_{t}^{1}-U_{t}|X_{p,t}^{1}-U_{p,t},X_{p,t}^{2}-V_{p,t}\right)p_{\mathrm{ref}}\left(X_{p,t}^{2}\right)dX_{p,t}^{2}}\\ & dX_{t}^{1}dX_{p,t}^{1}dX_{p,t}^{2}=\int p\left(X_{t}^{1},X_{p,t}^{1},X_{p,t}^{2}\right)\log\frac{p\left(X_{t}^{1}|X_{p,t}^{1},X_{p,t}^{2}\right)}{\int p\left(X_{t}^{1}|X_{p,t}^{1},X_{p,t}^{2}\right)p_{\mathrm{ref}}\left(X_{p,t}^{2}+V_{p,t}\right)dX_{p,t}^{2}}\\ & dX_{t}^{1}dX_{p,t}^{1}dX_{p,t}^{2}\neq \int p\left(X_{t}^{1},X_{p,t}^{1},X_{p,t}^{2}\right)\log\frac{p\left(X_{t}^{1}|X_{p,t}^{1},X_{p,t}^{2}\right)}{\int p\left(X_{t}^{1}|X_{p,t}^{1},X_{p,t}^{2}\right)p_{\mathrm{ref}}\left(X_{p,t}^{2}\right)dX_{p,t}^{2}}\;dX_{t}^{1}dX_{p,t}^{1}dX_{p,t}^{2}=\text{rDCS}\left(X_{t}^{2}\to X_{t}^{1}\right), \end{array}$$

because ${\tilde{p}}_{\mathrm{ref}}$ is not translated by the deterministic perturbation in the way $\tilde{p}\left(X_{p,t}^{2}\right)$ is (as the perturbation is happening after the reference time), such that the denominators do not allow equating the integrated terms by change of variables in the generic case. Therefore rDCS is capable of uncovering transient causal influences between stimulus-triggered events exhibiting a deterministic waveform.

### 2.5 Alignment for spontaneous events

The relevance of peri-event time-varying causal analysis using the proposed rDCS, as well as TE and DCS, depends on the modeling assumptions of peri-event data. In particular, we assume that the neural events we want to study reflect a sequence of continuously changing hidden states and that values at each peri-event time point *t*′ are sampled *i.i.d.* across trials from the same ground truth distribution (Shao et al., 2022) at *t*′. This is easily justified for stimulus-evoked events, as addressed in Section 2.3.2 and Section 2.4.2, with an intrinsic reference time for occurrence (i.e., the triggering time). However, analyzing spontaneous events whose occurrence times are not known *a priori*, such as transient events observed during sleep, requires 1) a *selection* procedure to identify them and 2) a procedure to choose a reference time point for each detected event, which is used to *align* all of them on a common *peri-event* time grid. The idea of reference points for alignment is similar to the anchor points in *Phase Rectified Signal Averaging* (Bauer et al., 2006). In contrast with such work, we focus on a transient phenomenon at the time scale of a peri-event time window instead of a very fast increase in the signal amplitude. Given a signal exhibiting spontaneous events, common procedures involve 1) *selecting* the events by thresholding a filtered version of this signal (that amplifies the events’ features of interest); 2) aligning events according to the local peak of this same filtered signal to best reflect the evolution of the underlying state. The result may only approximately recover the ground truth distribution of the events, as it is influenced by the choice of filtered signal and putative signal perturbations.

Importantly, selection may lead to a biased estimation of event statistics and peri-event dynamics, due to *selecting* data based on a specific detection signal, resulting in a misleading characterization of causal interactions (e.g., wrong causal directions as seen in Supplementary Figure S6B). We will thus study how event *selection* affects the estimation of causal influence and propose an appropriate procedure on this basis. To model the effect of selection, we use an SCM-based perspective on selection bias (Bareinboim and Pearl, 2012; Bareinboim et al., 2014). We can modify the SCM in Figure 2A to incorporate an additional node *S* representing the selection variable, which is a binary variable indicating whether the time window, with specified reference time point, is selected (Supplementary Section SC for background). Typically *S* is defined by testing whether a continuous random variable *D* goes over a predefined threshold. *D* is itself a function of the time series nodes within the peri-event time window, corresponding, for example, to the aforementioned filtering operation. A practical example is the detection of oscillatory events using a band-pass filter, where the dependency of *D* (and thus *S*) on other nodes reflects the dependency of the filtered signals on past samples of ***X*** through the coefficients of a causal Finite Impulse Response (FIR) filter.

**FIGURE 3:**
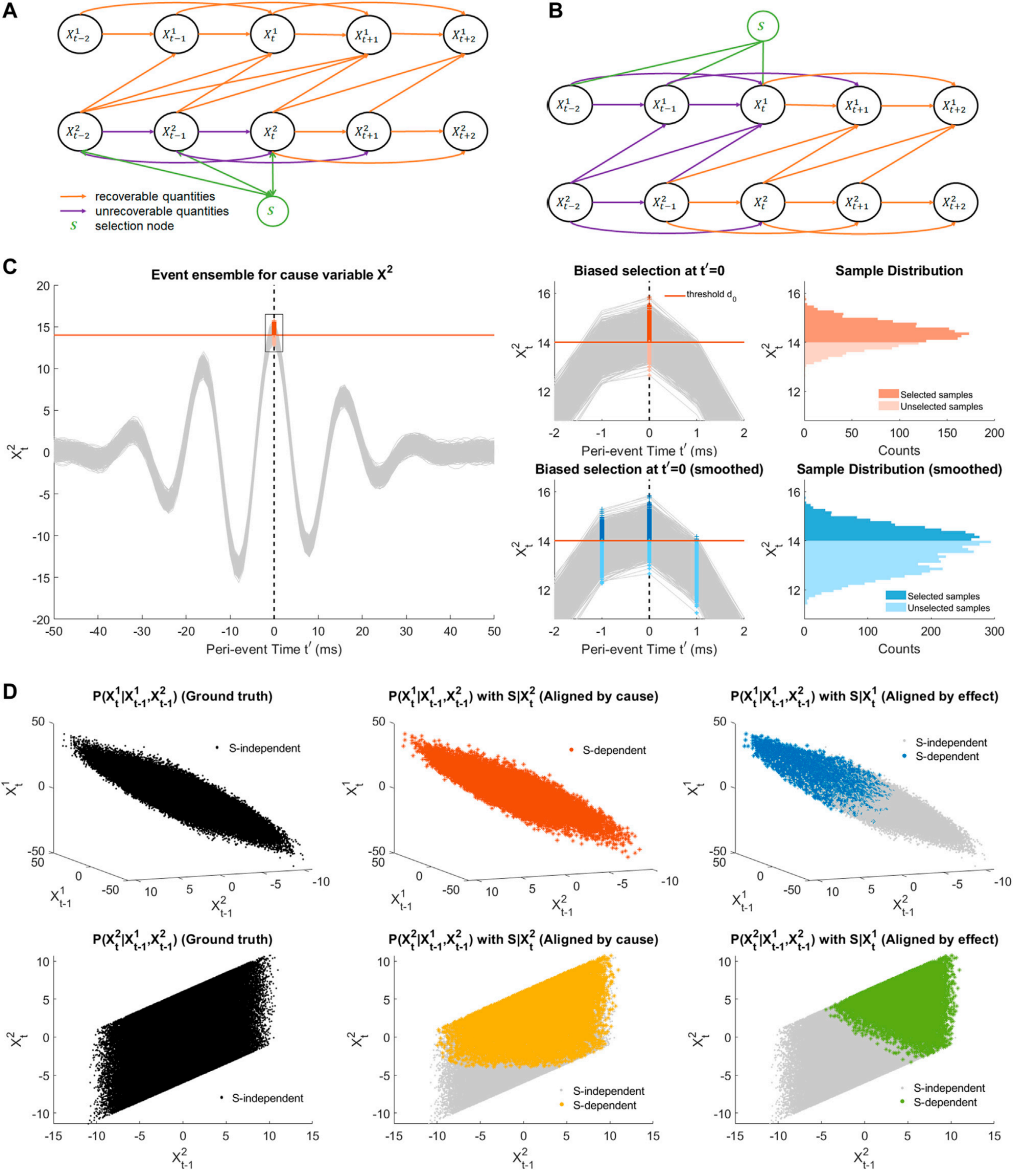
Illustration of selection bias due to thresholding and alignment. **(A)** SCM of a bi-variate SVAR(2) model with uni-directional coupling from *X* ^2^ to *X* ^1^ and a selection node S depending on states of the cause variable before peri-event time (*t*′< =0). The selection node S represents partial selection of samples due to thresholding of the filtered cause signal (as the detection signal). Orange arrows makes the recoverable arrows with the current selection node, while purple arrows indicates the unrecoverable ones. **(B)** The same SCM as in **(A)** with the selection node depending in a similar way on the effect signal. **(C)** An example event ensemble for the cause variable $X_{t}^{2}$ in **(A,B)** and the detection threshold. **(D)** Zoomed event ensenbles for **(C)** (left) and histograms for selected samples compared to the full sample (right). Top panel illustrates selection bias at ground truth peri-event time *t*′ = 0. The orange and shaded distributions represents histograms at a single time *t*′ = 0. Bottom panel shows selection bias at the peri-event time *t*′ = 0 for detected events aligned by the peak. The dataset aligned in this way reflects at *t*′ = 0 is a local average of the state trajectories in a neighborhood of the target ground truth state. **(E)** Illustration of recoverability when aligning by the cause. Subplots show joint distributions of the lagged variables and the putative effect variable of a SVAR(1) model with uniformly distributed innovations, with left column for the ground-truth alignment, middle column for aligning by the cause and right column for aligning by the effect. The conditional is only recoverable for the top middle panel. See Supplementary Figure S4 for the cases of peri-event time *t*′=0 and *t*′>0.

In practice, we can *a priori* choose *S* to depend either on the cause variables ***X*** ^2^ (Figure 3A) or on the effect variables ***X*** ^1^ (Figure 3B). Assuming that the filter (i.e., for constructing the continuous RV) is well chosen, and the selection threshold is high enough, choosing windows satisfying *S* = 1 will typically “over-select”, i.e., exclude some peri-event time series that would actually be relevant for our analysis. Figure 3C(left, top right) illustrates how thresholding selects only a subset of peri-event trajectory samples at *t*′ = 0 in a simulated scenario. This over-selection can then be modeled as sampling peri-event data from a conditional peri-event distribution *p*(*X*|*S*), while we are interested in analyzing a ground truth distribution *p*(*X*). This conditioning may induce a so-called *selection bias* in the estimation of quantities we are interested in, notably the conditional distributions that enter the calculations of TE, DCS and rDCS. The impact of such bias on those quantities as been investigated in Bareinboim and Pearl (2012); Bareinboim et al. (2014) within the SCM framework, as we describe in the following.

For simplicity and consistency with the Results section, we will restrict ourselves to models with a unidirectional causal effect (either *X* ^1^ → *X* ^2^ or *X* ^2^ → *X* ^1^) and assume that *S* is only dependent on a finite number of past peri-event times (*t*′ ≤ 0) as in the case of a causal FIR filter (for other cases, refer to Supplementary Section SC.2). Figures 3A, B illustrate in this setting that the conditional associated to causal arrow (*X* ^2^ → *X* ^1^) can be recovered at any peri-event time only when the selection node depends on the cause variable (Supplementary Section SC.2 for justification). Specifically, this means that $P\left(X_{t}^{1}|X_{p,t}^{1},X_{p,t}^{2},S\right)=P\left(X_{t}^{1}|X_{p,t}^{1},X_{p,t}^{2}\right)$ for the SCM in Figure 3A. For the opposite direction, $P\left(X_{t}^{2}|X_{p,t}^{1},X_{p,t}^{2},S\right)\neq P\left(X_{t}^{2}|X_{p,t}^{2}\right)$ for negative peri-event time *t*′ ≤ 0. For the case where *S* depends on the effect variable, $P\left(X_{t}^{1}|X_{p,t}^{1},X_{p,t}^{2},S\right)\neq P\left(X_{t}^{1}|X_{p,t}^{1},X_{p,t}^{2}\right)$ for negative peri-event time *t*′ ≤ 0 and $P\left(X_{t}^{2}|X_{p,t}^{1},X_{p,t}^{2},S\right)\neq P\left(X_{t}^{2}|X_{p,t}^{1},X_{p,t}^{2}\right)$ for *t*′ < 0 (see also Supplementary Section SC.2 and Supplementary Figure S4). The *S*-dependent and *S*-independent conditionals are visualized in Figure 3E for an example SVAR(1) model, as described in Section 2.3.2, where the innovations $\eta_{t}^{1}$ and $\eta_{t}^{2}$ are drawn from a uniform-distribution. Similarly, the conditional model of the post-intervention scenario for rDCS with selection node depending on the cause satisfies

$$\begin{array}{cc} p^{do\left(X_{t}^{1}:=f\left(X_{p,t}^{1},X_{p,t_{\mathrm{ref}}}^{2},\eta_{t}^{1}\right)\right)}\left(X_{t}^{1}|X_{p,t}^{1},X_{p,t}^{2},S\right) & =\int p_{\mathrm{ref}}\left(X_{p,t}^{2}\right)p\left(X_{t}^{1}|X_{p,t}^{1},X_{p,t}^{2},S\right)dX_{p,t}^{2}\\ & =\int p_{\mathrm{ref}}\left(X_{p,t}^{2}\right)p\left(X_{t}^{1}|X_{p,t}^{1},X_{p,t}^{2}\right)dX_{p,t}^{2}=p^{do\left(X_{t}^{1}:=f\left(X_{p,t}^{1},X_{p,t_{\mathrm{ref}}}^{2},\eta_{t}^{1}\right)\right)}\\ & \left(X_{t}^{1}|X_{p,t}^{1},X_{p,t}^{2}\right) \end{array}$$

Therefore, the KL divergence for the ground truth direction *X* ^2^ → *X* ^1^ can be estimated correctly when selecting the event based on the ground-truth cause variable “*S*(*X* ^2^)”, while this does not hold for the opposite direction *X* ^2^ → *X* ^1^ nor when selecting based on the ground-truth effect “*S*(*X* ^1^)”. As the true causal direction is unknown, we thus propose that, to investigate the dominant causal direction between two event ensembles, we should focus on comparing the causality measures (TE, DCS and rDCS) for each direction when the events are aligned on the putative cause, i.e., *X* ^2^ → *X* ^1^|*S*(*X* ^2^) compared to *X* ^1^ → *X* ^2^|*S*(*X* ^1^). Although rDCS is expected to be biased for the second case when aligned by the effect variable, for uni-directionally coupled systems, as the ground truth rDCS is zero, we expect the bias to still lead to a comparatively small rDCS value relative to ground truth, such that the contrast between two directions is preserved.

Other factors may affect the estimation of causal strength. Since rDCS is defined as the expectation over the KL divergence over the past states $X_{p,t}^{1}$ and $X_{p,t}^{2}$ (Eq. 11), reliable estimation of rDCS $\left(X_{t}^{2}\to X_{t}^{1}\right)$ also depends on the unbiased sampling of the joint probability of $X_{p,t}^{1}$ and $X_{p,t}^{2}$ . We argue here that this is approximately satisfied as the conditioning is made on a specific detection signal rather than on these variables, such that they are mildly affected by it.

Next, the above mentioned alignment procedure may affect causal strength estimation. *Perfect alignment* (considered *ground truth*) refers to the condition where the ground-truth hidden states are identical for all trials at each peri-event time *t*′ in an extracted event ensemble, as shown in Figure 3D for *t*′ = 0. In this scenario, no further alignment is needed as all trials are intrinstically aligned. In order to study the influence of the aforementioned selection bias specifically due to thresholding, we may still apply selection to the perfectly aligned dataset, resulting in excluding below threshold samples from the estimation procedure. We refer to this situation as *single-time selection* of events where trials are aligned based on known ground-truth reference time. This setting assumes that one knows the hidden state, which is possible only for stimulus triggered or simulated events, but impossible for experimentally observed spontaneous events. In the latter case, by thresholding over the whole observed signal one typically end up selecting successive sliding time windows that all have a detection signal exceeding the threshold (e.g., Figure 3D(Bottom right)). Selecting all these points can be interpreted as smoothing the ground truth state over all these neighboring state space points, an alignment scenario which we name as *smoothed alignment* of events. In practice, a common alternative is to further select among above-threshold overlapping peri-event time-windows the local peaks as the reference points, which can be understood as a non-uniform subsampling of the smoothed alignment and can be unified into the same scenario category.

### 2.6 Data processing pipeline

The whole analysis procedure can be conducted in two phases: event selection and causal analysis. We will elaborate on the detailed steps in each phase in the following.

• Phase 1: Event Selection
  1. *Filtering*: given a bi-variate signal (as a simple case), for different purposes of study, one would need to find an appropriate filter to apply to the original signals such that certain features of the underlying system can be amplified. For example, to locate the Sharp Wave-Ripples (introduced in the Introduction and analyzed in Section 3.3) that are prominent in the ripple band [80–250]Hz, one would use a bandpass filter such that the irrelevant components are attenuated. Events can be also detected with a template matching procedure, which is another type of filtering (Supplementary Figure S2).
  2. *Thresholding*: a certain threshold is determined beforehand (up to the specific feature of the question) and applied to the filtered signal. As the filtered signal is designed to amplify the feature, time points where the filtered signals are over the threshold are candidate reference points. Reference points define the peri-event time t’ and are used to extract peri-event data as multiple trials.
  3. *Alignment*: the thresholding procedure can be applied to either the cause or effect signals. One can select all candidate reference points obtained by filtering either signal (for the smoothed alignment case) or the time points of local peaks (of the filtered signal) as reference points. Then the bi-variate peri-event trials are extracted in a fixed-length window surrounding the reference points, thus forming the peri-event ensemble for further analysis.
• Phase 2: causal analysis
  1. *Model order selection*: as mentioned in Section 2.2.1, our estimation of information theoretic quantities is based on time-inhomogeneous SVAR models. One thus needs to determine the optimal SVAR model order that best reflects the underlying dynamics. A common approach for model order selection is the Bayesian Information Criterion (BIC), which we have extended to the time-varying case in Shao et al. (2022) using the extracted event ensembles obtained in the first phase.
  2. *SVAR model estimation*: Shao et al. (2022) also provide a way to estimate the SVAR model with the extracted event ensemble and the optimized model order. Thus we will obtain an estimate of the autoregressive parameters, i.e., the autoregressive coefficients and innovation mean and variance.
  3. *Computation of causality measures*: with the estimated autoregressive parameters and the signals second order statistics, we can estimate the time-varying causality measures as detailed in Supplementary Section SD: TE based on Supplementary Equation S8, DCS on Supplementary Equation S9 and rDCS on Supplementary Equation S10.

Notably, the causal analysis procedure can be applied to event ensembles obtained with any type of alignment. However, as elaborated in Section 2.5, we propose to compare the causality measures in two different directions from the event ensembles where trials are aligned by the putative causes. To facilitate the application of this analysis framework, we have made available the code that performs the aforementioned experimental procedure (see https://github.com/KaidiShao/event_causality_frontiers).

## 3 Results

In this section, we first focus on illustrating the properties of TE, DCS and rDCS with simulated toy models. The problem of vanishing TE occurring with synchronized signals and the benefits of DCS in the same situation will be investigated in Section 3.1. Next, we simulate a simple uni-directionally coupled SVAR(4) system with rhythmic perturbations of the cause variable to generate transient events, where we will show that rDCS is able to reflect the change of causal effects due to this perturbation while TE and DCS fail. We also study the influence of the alignment method in the same example, as well as in experimental *in vivo* recordings from uni-directionally coupled hippocampal regions during SWRs.

### 3.1 The case of strongly-correlated signals

As mentioned in Section 2.3.1, TE does not capture well causal influences when the cause and effect signals are strongly correlated with each other, contray to DCS. Here, to illustrate such contrast, we simulate a bivariate dynamical system in the form of two synchronized continuous harmonic oscillators *x*(*t*) and *y*(*t*), with uni-directional coupling (i.e., *x*(*t*) driving *y*(*t*)):

$$\left\{\begin{array}{cc} \frac{d^{2}x}{dt^{2}} & =-2\zeta_{x}\omega_{x}\frac{dx}{dt}-\omega_{x}^{2}x+n_{x},\\ \frac{d^{2}y}{dt^{2}} & =-2\zeta_{y}\omega_{y}\frac{dy}{dt}-\omega_{y}^{2}y+cx+n_{y}. \end{array}\right.$$ (13)

In this system, *x*(*t*) is designed as an under-damped oscillator (*ζ*_*x*_ = 0.015722), which approximately oscillates at a period *T*_*x*_ = 200 samples corresponding to natural (angular) frequency *ω*_*x*_ = 2*π*/*T*_*x*_ = 0.0314 rad/sample. To achieve synchrony, *y*(*t*) is also designed as an under-damped oscillator (*ζ*_*y*_ = 0.2) whose intrinsic oscillation gradually vanishes and finally follows the oscillation of *x*(*t*) with a coupling strength of *c* = 0.098. For *y*(*t*), *T*_*y*_ = 20, *ω*_*y*_ = 2*π*/*T*_*y*_ = 0.314. We also add small Gaussian innovations to both oscillators: $n_{x}∼N\left(0,0.02\right)$ , $n_{y}∼N\left(0,0.005\right)$ . Adding this noise allows fitting a SVAR model to the signals to assess the causal interactions with TE and DCS. SVAR parameter estimation would fail with deterministic signals by causing the covariance matrix estimates to be singular.

Using the Euler method with a time step of 1 and random initial points (N(0,1)) , we simulated 2000 trials of this uni-directionally coupled system with 1000-point length. We discarded the first 500 points to ensure that the time series reach a sufficient level of synchronization. We can see this system as a stationary SVAR(2) process because numerical simulation with the Euler method generates data as a function of the last two past states. The idea of using a SVAR(2) model is elaborated on in the Supplementary Section SE. Notably, modeling simulated data with a SVAR(2) model is also possible if the numerical integration method is switched to Runge-Kutta, despite the SVAR(2) parameters having a more complex form than the continuous formulation of the system.

**FIGURE 4:**
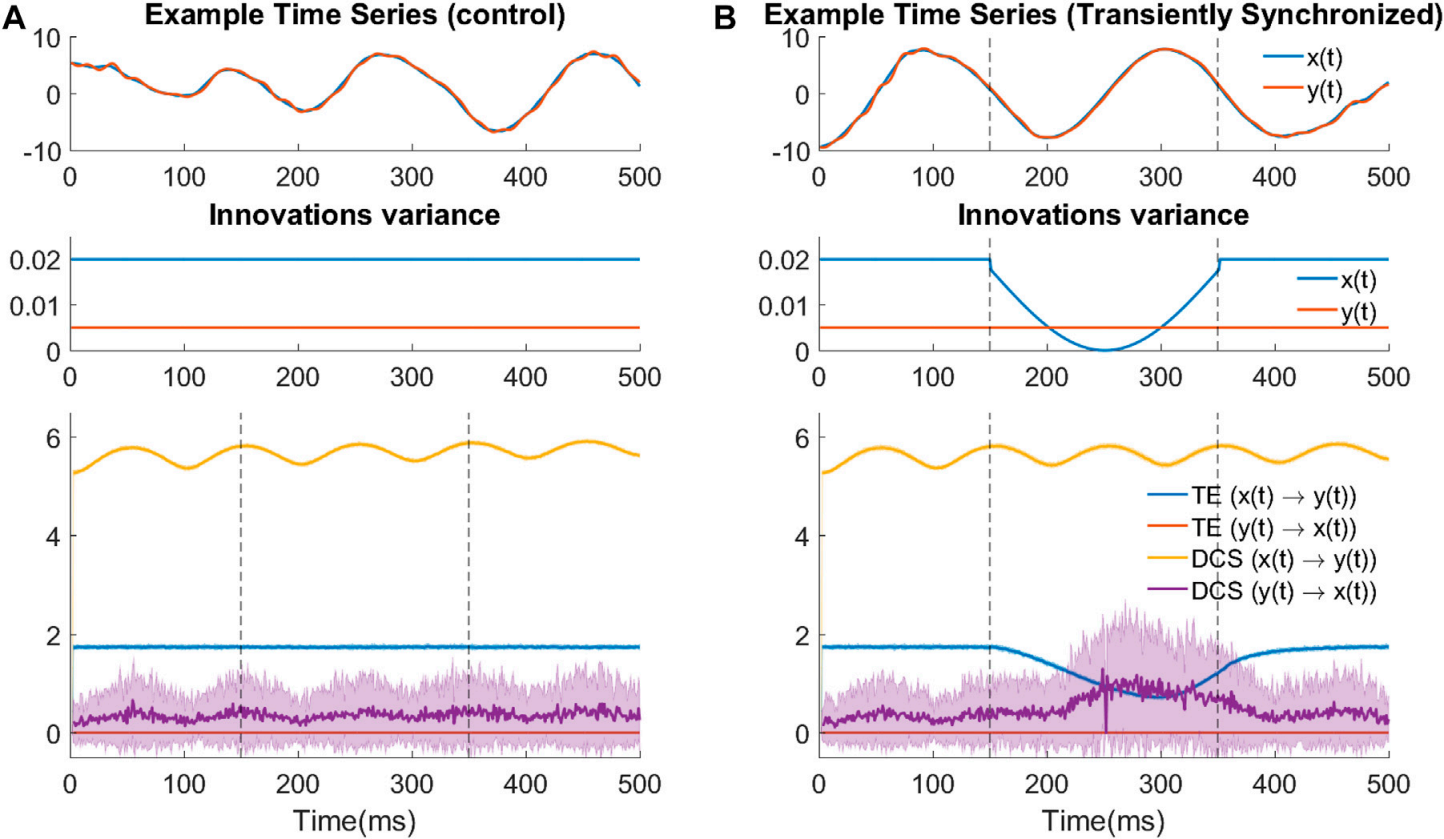
TE fails when the signals are strongly synchronized. **(A)** Control experiments where synchrony is not changed. (top) Example trace of the bivariate signal in the control experiment. (middle) Time-varying design of innovation’s variance for both variables in the control experiment. (bottom) Time-varying TE and DCS results in the control experiment. **(B)** TE underperforms during transient increased synchrony induced by a tiny change in noise variance. The transient change can be seen as an event. Subfigure designs are the same as **(A)**.

Figure 4A shows the results of time-varying TE and DCS for assessing the causal effects between *x*(*t*) and *y*(*t*). Calculation is performed in both the ground truth direction (*x*(*t*) → *y*(*t*)) and the opposite direction. We first look at the control experiment. Consistent with the system’s stationarity, TE is constant in both directions while being higher in the ground-truth direction. DCS in the ground-truth direction stays at a relatively high level, despite some small oscillation under a frequency similar to the intrinsic oscillation frequency of *x*(*t*).

With respect to the detection of causal direction, both measures are able to detect the correct direction (i.e., causation for *x*(*t*) → *y*(*t*) is much larger than in the opposite direction). It is also reasonable that DCS in both directions is higher than TE, according to its definition in section 2.2.3. However, from the control experiment, we cannot conclude that the smaller TE values are due to its definition or due to the strong synchrony in the signals.

Therefore, we introduced a transient decrease of the noise variance in the cause signals (*x*(*t*)). The logic of designing this transient change is the following: the level of synchronization will increase with weaker noise, but the system and input magnitude remain the same because the contribution of the noise change to the signal amplitude is negligible; thus if TE is insensitive to the level of synchronization of signals, its values are expected to stay constant. However, as the results show in Figure 4B, there is a transient decrease of TE during the interval where noise variance is decreased, suggesting that TE performs poorly in the cases where the cause and effect signals are strongly synchronized. As such strong synchronized oscillations are common phenomena in the context of transient neural events, one would need to pay extra attention when using TE (as a widely-applied causality measure) to investigate the direction of causation during these transient phenomena.

### 3.2 The case of deterministic perturbations

In this section, we directly address the benefits of rDCS over TE and DCS when applied to signals driven by deterministic perturbations. To illustrate this specific property, we designed some simple transient events perturbing the innovation parameters of a stationary SVAR process with uni-directional coupling. The events are generated by feeding the cause signal with innovations with non-zero time-varying means, such that both signals will exhibit temporal oscillations. We refer to these events as *perturbation events* in the following sections. These perturbations intrinsically define a hidden state that parametrizes the ground truth distribution of peri-event data. We exploit the hidden state and demonstrate that the proposed alignment method in Section 2.5 is efficient for recovering the time-varying causal direction between the two variables.

#### 3.2.1 Simulation procedure

We simulated a non-stationary uni-directionally-coupled autoregressive system defined in Eq. 2a and Supplementary Eq. 2b. The causal direction is *X* ^2^ → *X* ^1^. The system is designed as a bivariate SVAR(4) process with a time-invariant coefficient matrix: **a** ^*⊤*^ = [−0.55, − 0.45, − 0.55, − 0.85], **b** ^*⊤*^ = [1.4, − 0.3, 1.5, 1.7], **c** ^*⊤*^ = [0, 0, 0, 0] and **d** ^*⊤*^ = [0.9, − 0.25, 0, 0.25]. These coefficients were randomly generated and kept after checking the stability of the SVAR(4) system. Uni-directional interactions are ensured by setting the autoregressive coefficients associated to interactions in the opposite direction (i.e., **c**) to zero for all lags.

**FIGURE 5:**
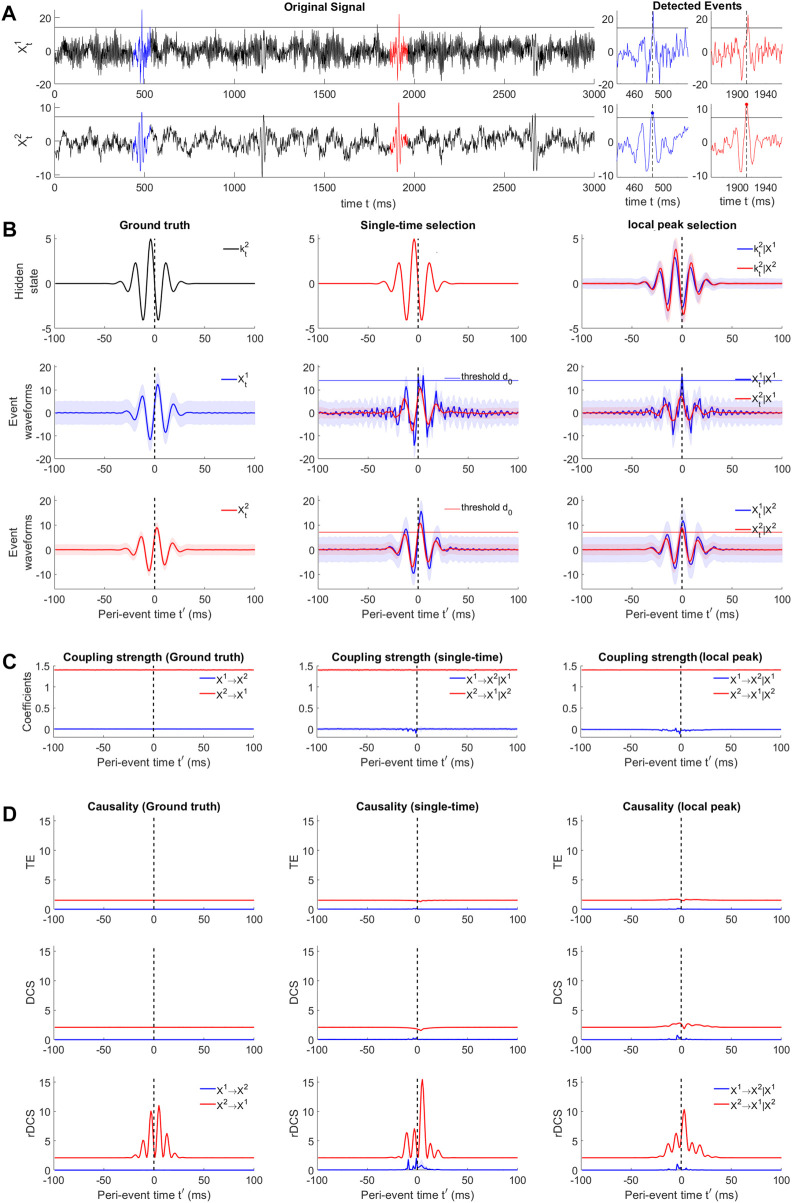
Causal analysis for simulated perturbation events with non-zero innovations. **(A)** Example signal traces of the bi-variate SVAR(4) system (black). Blue and red traces mark two example events detected by thresholding over the cause $X_{t}^{2}$ . Blue and red dots show other reference points. **(B)** (Top) Hidden states for *ground-truth alignment* (left), *single time selection* of the ground truth event ensembles due to thresholding (middle) and events aligned by local peaks over threshold (right). (Middle) ground truth event ensemble for $X_{t}^{1}$ (left) and bi-variate ensembles of the other two selections aligned by $X_{t}^{1}$ (middle, right). Thin blue line represents the threshold in $X_{t}^{1}$ . (Bottom) Same settings as in (middle) but aligned by $X_{t}^{2}$ . **(C)** Example elements of coupling strength in the ground truth directions $X_{t}^{2}\to X_{t}^{1}$ (red) and the opposite direction $X_{t}^{1}\to X_{t}^{2}$ (blue) for 3 types of event ensembles aligned by putative cause. **(D)** TE (left), DCS (middle) and rDCS (right) for all 3 types of event ensembles aligned by putative cause.

We enforce non-stationarity of $\eta_{t}^{2}$ , the innovations of the ground truth cause process $\left\{X_{t}^{2}\right\}$ . Both innovations $\eta_{t}^{1}$ and $\eta_{t}^{2}$ are drawn from a Gaussian distribution with unit variance (with no correlation in between, i.e., $Cov\left[\eta_{t}^{1},\eta_{t}^{2}\right]=0$ ); the difference is that $E\left[\eta_{t}^{1}\right]=k_{t}^{1}=0$ while $E\left[\eta_{t}^{2}\right]=k_{t}^{2}$ is non-zero and time-varying. We designed the time-varying profile of $k_{t}^{2}$ as a Morlet-shaped waveform to mimic the oscillatory properties of neural event signals: $k_{t}^{2}=H\exp\left(-{\left(\alpha x\right)}^{2}/2\right)\cos\left(5\alpha x\right)$ , where *α* = 2/25 is a constant controlling the event duration, and *H* = 4 is the amplitude of the highest peak in the center of the event. The total duration of the Morlet-shaped waveform is 101 ms. The innovation’s mean designed for *X* ^2^ is shown in Figure 5B (top left panel).

We generated this bi-variate SVAR(4) process for 1300*s* consisting of 5,000 trials of perturbation events by transiently varying $\eta_{t}^{2}$ , detecting event occurrence based on the cause $X_{t}^{2}$ , as illustrated in Figure 5A. The central peaks of these Morlet events are used as the ground-truth reference points for which peri-event time *t*′ = 0, and used to extract a dataset of multi-trial events ensemble with a 200-*ms* peri-event window such that *t*′ ranges, from −99 *ms* to +100 *ms* (i.e., there is no alignment procedure that could lead to selection bias, see Section 2.5). The event waveforms of the cause variable $X_{t}^{2}$ and the effect variable $X_{t}^{1}$ are illustrated in Figure 5B (bottom left, middle left). The whole process is repeated 100 times to obtain variabilities plotted in the figure.

#### 3.2.2 Effect of trial selection and alignment on model estimation and causality measures

The designed deterministic innovation (i.e., identical across trials), can be seen as imposing a hidden state evolving across the peri-event interval. The event ensembles obtained by this ground truth model define a dataset where no event selection and alignment is needed. We can compare the SVAR model estimation and causality measures resulting from this dataset to the outcomes obtained by selecting and aligning events based on either variable *X* ^2^ or *X* ^1^, as discussed in Section 2.5.

To validate the recoverability theory in the presence of selection bias due to the event detection procedure, we test the *single-time selection* setting (see Section 2.5) where sub-threshold trials are removed from the ground truth peri-event dataset (as illustrated in Figure 3D(Top right)), thus preserving the ground-truth hidden states (Figure 5B(Top middle)). The peri-event trials having reference point values higher than a threshold *d*_0_ = 3*SD* for the chosen variable are selected, where the standard deviation is computed from the whole signal. The selected event ensembles are shown in Figure 5A(middle center) for thresholding based on $X_{t}^{1}$ and Figure 5A(middle right) for thresholding based on $X_{t}^{2}$ . Notably, this kind of selection is only feasible when the hidden state in known, which is not realistic practically for real data.

Next we demonstrate the appropriateness of the approach performed on real data (i.e., selection and smoothed alignment based on putative cause), we set *d*_0_ as a threshold and performed smoothed alignment over the original signal itself. We obtain an event ensemble by selecting local peaks for points over *d*_0_ as new reference points, which is shown in Figure 5B(middle right and bottom right). This can be seen as a smoothed version of the ground-truth dynamics, which is also confirmed by checking the aligned hidden states (Figure 5B(Top right)).

While inferring SVAR model parameters of the event ensembles according to Shao et al. (2022), the true model order 4) can be recovered for all five ensembles. Figure 5C demonstrates the recoverability of conditional probabilities for ensembles aligned by the putative cause. Coupling strengths from the putative cause to the putative effect are plotted in red. As described in the simulation procedure in Section 3.2.1, the coupling strength is constant over time, which is reflected in Figure 5C(left). Consistent with the theory in Section 2.5, biased selection of event trials on the samples at *t*′ = 0 leads to unbiased estimation of the coupling strength $X_{t}^{2}\to X_{t}^{1}$ aligned by the cause *X* ^2^ (denoted also as “∣*X* ^2^” in Figure 5C(middle)). By comparison, the coupling strength in the other direction is slightly biased at negative peri-event times (*t*′) but still relatively close to its true value 0). This contrast holds for alignment with local peaks over threshold, as seen in Figure 5C(right).

Figure 5D (Top, middle, bottom) shows the corresponding results of how causality measures perform in the three alignment scenarios. For clearer visualization of TE and DCS with a zoomed vertical scale, see Supplementary Figure S8. During the periods where no transient events occur, all three measures are able to infer a time-invariant causal effect in the ground-truth direction (*X* ^2^ → *X* ^1^) compared to the opposite direction. Besides, in line with theoretical predictions, DCS is higher than TE and is equal to rDCS. During the perturbation events, in the ground truth direction TE and DCS remain constant and rDCS exhibit a rhythmic pattern. These results match the theoretical predictions: TE and DCS measures the connectivity strength, which does not change, while rDCS measures the combined causal effect related to the connectivity and the event-based changes at the cause, yielding larger variations transmitted to the effect node.

In the transient time scale, thresholding leads to selection bias in estimating causality measures. In the case where event ensembles are aligned by *single-time selection* of the cause $X_{t}^{2}$ , TE and DCS of the ground truth direction is underestimated while rDCS is slightly overestimated around *t*′ = 0. A bias appears in the opposite direction while aligned by the effect, but the direction of causation is detected correctly. The case of local peak alignment shows similar results, except the peak amplitude of the smoothed rDCS is less amplified. Notably, in the smoothed case, a transient increase is observed in both TE and DCS, resembling the envelope of the perturbation. This is likely an effect of the smoothing procedure but is quickly interrupted by a negative bias due to thresholding, making the results unreliable in detecting transient changes. We also showed a negative example with putative effect alignment in Supplementary Figure S5, where the coupling strength in the causal direction is much weaker than the model in Figure 5. The coupling strength of the causal direction undergoes a sharp decrease at peri-event time *t*′ = 0, leading to a transient underestimation of TE, DCS and rDCS for both single-time and local peak scenarios. The close-to-zero value of rDCS is misleading for the inference of transient causal interactions, thus illustrating the unreliability of putative effect alignment.

Thus, this simulation experiment of perturbation events demonstrates the effectiveness of rDCS in reflecting the causal influence when the cause is perturbed by a deterministic exogenous input compared to TE and DCS, validating that rDCS is a better measure to address event-based causal interactions. More importantly, we highlight here the trial alignment problem when dealing with event-based data, especially when events occur spontaneously. Supplementary Figure S7 is a clear example showing the impact of alignment on information-theoretic measures: aligning on the actual effect could reverse the detected direction of causation. Thus, by contrasting the different impacts of alignment on information-theoretic measures, we show that in practice, selection via thresholding and aligning the event ensemble with the local peaks of the putative cause is a good way to assess the ground truth event-based causality given uni-directional connections. This approach will be further applied to real data in the next section.

### 3.3 Validation on SWRs-based causality between CA3 and CA1 regions

Sharp Wave-Ripple (SWR) events, hypothesized as a key element in implementing memory consolidation in the brain, have been reported in the electrophysiological recordings within the hippocampus of both macaques and rodents. In this section we detect SWRs in an experimental dataset to investigate the behavior of TE, DCS and rDCS in a neuroscientific context where the event-hosting brain regions are uni-directionally coupled, i.e., in a situation where the causal direction is known *a priori*.

SWRs are primarily generated in the CA1 area of the hippocampus. The somas of CA1 pyramidal cells are located in the pyramidal layer (‘pl’) while their dendritic trees are rooted in the stratum radiatum (‘sr’). It is hypothesized that the dendritic trees receive strong excitatory inputs from the pyramidal cells in CA3 which generate post-synaptic activities in the dendritic trees. This results in LFP activities at low frequencies (0–30Hz, due to the sharp-wave) and in the gamma band (30–80Hz, due to CA3 oscillations). Then the dendritic activities propagate to the soma, where recurrent interactions between inhibitory and excitatory cells generate a fast oscillation, the ripples (80–250 Hz).

**FIGURE 6:**
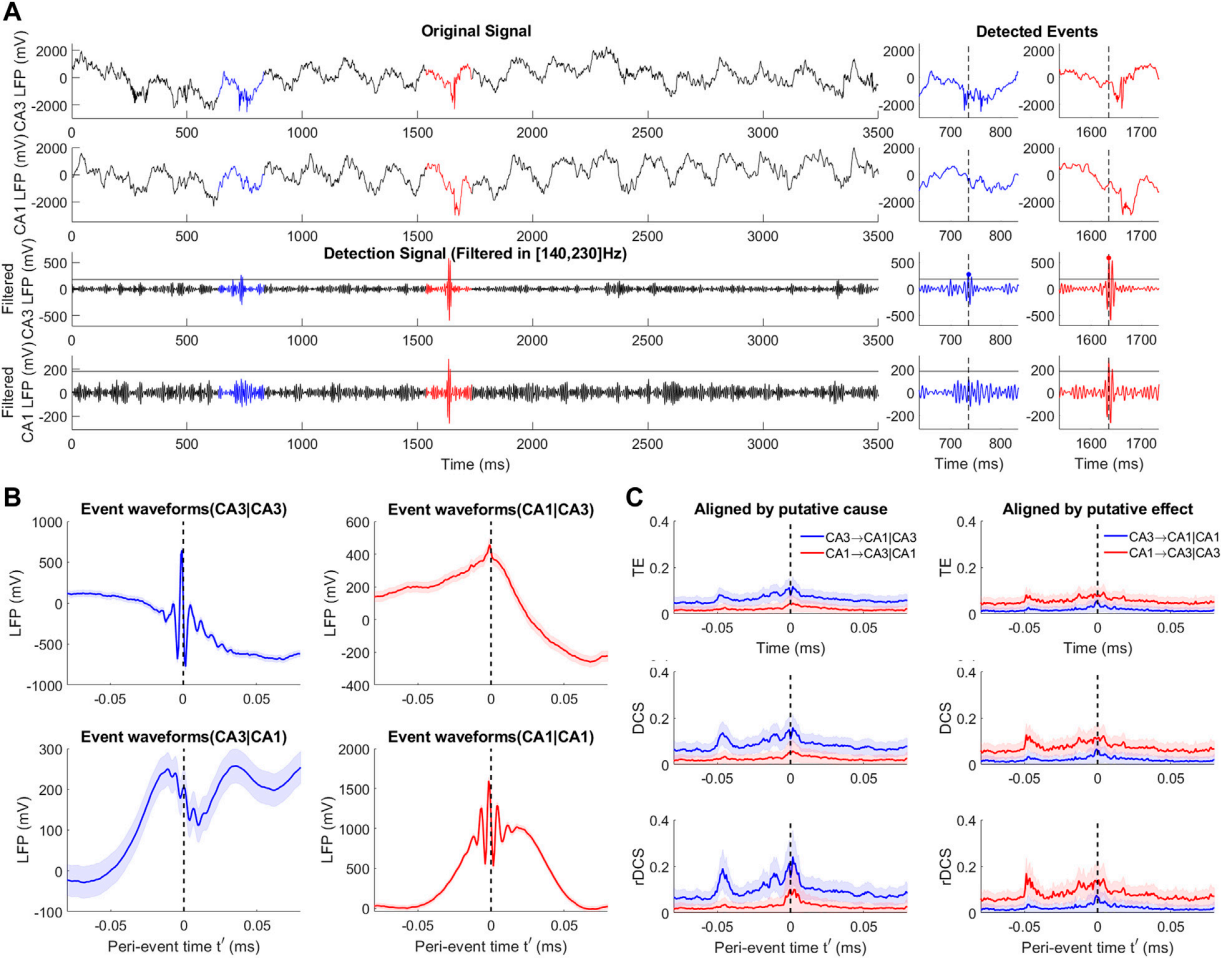
Event-based causal analysis for SWRs in rodent hippocampal CA3 and CA1 regions. **(A)** Examples signal traces of the original signals and bandpass filtered signals of CA3 and CA1 regions (black). Blue and red traces mark two example events detected by thresholding over the cause CA3 and aligned by the local peak. Blue and red dots show other reference points. **(B)** Event waveforms of SWR event ensembles at CA3 (left) and CA1 (right) regions aligned by CA3 (Top) and CA1 (Bottom) signals. Shades repensent the ensemble standard error averaged over 1024 channel pairs. **(C)** Peri-event causality measured by TE (Top), DCS (middle) and rDCS (Bottom) for event ensembles aligned by the putative cause (left) and the putative effect (right). Shades reflect standard deviation of 100 repeated bootstrapped ensembles.

We applied the event-based causality analysis to an open source dataset where electrophysiological recordings in the CA3 and CA1 regions of rodent hippocampus have been performed with 4 shanks of 8 channels simultaneously in each region (Mizuseki et al., 2014). In agreement with the SWR generation mechanism explained in the above paragraph, anatomical studies (Csicsvari et al., 2000) support uni-directional anatomical coupling between these two regions within the hippocampal formation, i.e., the ground truth direction is known to be CA3 → CA1. The analysis is based on two Local Field Potential (LFP) data sessions recorded from the rat named ‘vvp01’ with a sampling rate of 1252 Hz. An example trace of a channel pair of both CA3 and CA1 regions is shown in Figure 6A. As SWRs are more challenging to observe during behavioral sessions, we perform our analysis only on a session of sleep which lasts 4943.588*s*.

Following Mizuseki et al. (2009), we detect SWRs by applying an 49-ordered FIR filter in the frequency band [140, 230]Hz to each channel of signals in both regions. The detailed detection procedure has been elaborated in Section 2.6 for the reference of readers and is similar to what is performed in Section 3.2. We set a threshold over the mean of the filtered signals (5 SD) to locate the events and align them according to the local peak time points over threshold.

Figure 6A(Bottom) shows the case aligned on the CA3 signals. The peri-event window for display has been chosen to be [-79.9, 79.9]*ms*, while VAR model estimation and the BIC-based model order selection are performed according to Shao et al. (2022). For each channel pair, we obtain two bi-variate event ensembles, corresponding to the two alignment conditions; thus, in total, we extract 2*1024 event ensembles (1024 channel pairs and 2 alignment conditions). The event waveforms and statistics of an example channel pair for different alignments are illustrated in Figure 6B.

SWR-based causality measures shown in Figure 6C compare the alignment by the putative cause and by the putative effect. The reference states used for estimating rDCS are the averaged states over the first 16 *ms* time points in the window. The standard deviation plotted in the figure originates from 100 times bootstrapped ensembles and the variability is averaged over 1024 channel pairs. In line with the theoretical predictions, the ground truth direction (CA3 → CA1) is well recovered when using an alignment by the putative cause, but not when aligning by the putative effect. TE, DCS and rDCS in the opposite of the truth direction are not significantly different from zero, which is consistent with the uni-directionality of anatomical connections posited by anatomical studies. Significantly stronger causal influences in the ground truth direction are shown by TE, DCS and rDCS before the alignment point (*t*′ = 0), matching the hypothesized SWR generating mechanism that the CA3 region drives the SWR interactions in CA1 region. The lack of difference between the two directions in more stationary states might be explained by the ineffectivity of causal measures based on linear VAR models to capture non-linearity (Shajarisales et al., 2015). The transient increase in the non-ground truth direction when using alignment on the putative cause might be explained by the selection bias elaborated on in Section 2.5.

## 4 Discussion

In summary, we have discussed the benefits and shortcomings of two time-varying causality measures (TE and DCS) in characterizing causal interactions based on peri-event data. To address their insensitivity to deterministic perturbations, we proposed a novel measure, rDCS, justified within the SCM framework. We compared the performance of these causality measures on perturbation events with innovations having time-varying means and electrophysiological recordings of hippocampal SWRs. The benefits of rDCS are supported by the perturbation events presented in Section 3.2. As causality analysis of transient events aims at uncovering the network mechanisms underlying these phenomena (e.g., addressing whether one event *drives* the other), we argue for the use of rDCS as it provably captures causal influences due to event-related changes in the cause that propagate to target regions through anatomical connections, even if these changes have little variability across trials. The outcome of rDCS is further illustrated on *in vivo* recordings of SWRs events in two hippocampal subfields.

Transient events are nonstationary signals that likely occur when the brain undergoes a transition from one state to another. Studying the “local” properties of the underlying non-equilibrium dynamics in regions of the state space might provide insights into the mechanism driving this transition. Earlier methods investigating such local dynamic properties include the local Lyapunov exponent (Pikovsky, 1993), while other common methods characterize local interactions between state variables within a short sliding time window, e.g., the local cross correlation (Buchner et al., 2009) or piecewise Granger causality (Ding et al., 2006). Our approach, although focused on the meaningful quantification of causal strength, is in line with the latter idea, where the time-varying SVAR model finds a 1-step local linear mapping in the trajectory formed by event trajectories, thus enabling to reveal transient causal interactions at a fast time scale, which may differ from the results obtained at equilibrium. As the measures are based on SVAR models, they can also be easily extended to a spectral form in order to capture the rich spectral properties in transient dynamics.

Contrasting the three measures of causal strength, TE is designed to assess conditional dependencies in observational data, while DCS and rDCS exploit this information to infer the impact of performing interventions of the SCM. In theory and as shown in the experiment of Section 3.1, TE can lead to counterintuitive outcomes applied to strongly synchronized events (a widely observed nonlinear phenomenon). While support has been provided for DCS and rDCS to be more appropriate measures of causal strength, they still require, like TE, certain assumption to be met (see also Section 2.1). A major concern is unobserved confounding, which might bias the estimated causal directions (e.g., the Simpson’s paradox in Pearl (2000)). Confounding effects can be corrected for by including activities from other regions, and there are also a few theoretical approaches to account for unobserved confounding under strong assumptions (Geiger et al., 2015; Mastakouri et al., 2021).

Selection bias is a fundamental issue for analyzing spontaneous neural activities, especially in case of any unsupervised detection or analysis. In this study we have demonstrated its impact on the alignment of the detected transient events and the resulting bias in causal inference. However, our proposal of *putative cause alignment* to estimate causal effect is theoretically supported only in the case of uni-directional coupling. Future work should assess the effect of selection bias in the case of bidirectional interactions and establish a framework to correct for such bias, not only in the context of causal strength inference but more generally for recovering the underlying event dynamics.

## Data Availability

Publicly available datasets were analyzed in this study. This data can be found here: https://github.com/KaidiShao/event_causality_frontiers, https://crcns.org/data-sets/hc/hc-3/about-hc-3.

## Footnotes

1 Because initial conditions of this deterministic linear system are set to zero before the intervention at *t*_0_
