# Supplementary material for: Information theoretic measures of causal influences during transient neural events
Source: Front Netw Physiol. 2023 May 31;3:1085347. doi: 10.3389/fnetp.2023.1085347 (PMC10266490; doi:10.3389/fnetp.2023.1085347)
Supplement: Supplementary file 1 [file DataSheet1.pdf]

Supplementary Material

CONTENTS

A Structural causal models 2
- A.1 Definition and properties 2
- A.2 Interventions 3

B Proof of insensitivity of DCS to deterministic perturbations 4

C Selection bias 4
- C.1 Recoverability with Sampling Selection Bias 4
- C.2 Recoverability with Sampling Selection Bias in VAR(p) models 5

D Derivations for time-varying causality measures 6
- D.1 Time-varying VAR(p) model 6
- D.2 Derivation of KL-divergence between two uni-variate Gaussians 8
- D.3 Conditional mean and variance for the actual condition 9
- D.4 Transfer Entropy 9
- D.5 Dynamic Causal Strength 10
- D.6 Relative Dynamic Causal Strength 11

E Dynamical system as a SVAR(2) model for numerical simulation 12
- E.1 Euler's method 12
- E.2 Runge-Kutta method 12

F Additional experiment for putative effect alignment 14

LIST OF FIGURES

S1 3
S2 5
S3 6
S4 7
S5 15
S5 16
S6 16
S7 17
S8 18

A STRUCTURAL CAUSAL MODELS

A.1 Definition and properties

We introduce first important tools to address questions pertaining to causality. Interestingly, these tools apply to probabilistic models that do not necessary include time information. This framework relies on structural equations of the form

$$Y := f(X_1, \cdots, X_k, \epsilon)$$

where the right hand side determines the assignment of values on the left-hand side. In the most usual case, Y and $\{X_j\}_{j\in\{1,\cdots,k\}}$ represent observed variables and ϵ a variable accounting for (unobserved) exogenous effects. Combining structural equations allows building Structural Causal Models (SCM) that describes dependencies between multiple observed variables.

DEFINITION 1 (Structural Causal Model (SCM) (see e.g. (Peters et al., 2017))). *A d-dimensional structural causal model for d endogenous variables $\{V_j\}_j = 1..d$ is a triplet $(\mathbb{S}, P_N, \mathcal{G})$ consisting of:*

- *a directed acyclic graph $\mathcal{G}$ with d vertices*
- *a set $\mathbb{S}$ of structural equations*

$$V_j := f_j(\boldsymbol{PA}_j, N_j), j = 1, \dots, d,$$

where $\boldsymbol{PA}_j$ are the variables indexed by the set of parents of vertex j in $\mathcal{G}$

- *a joint distribution P_N over the exogenous variables N_j, which are assumed mutually independent.*

EXAMPLE 1. *Take the following linear SCM*

$$X := N_1,\ N_1 \sim \mathcal{N}(0, 1), \tag{S1a}$$

$$Z := N_2,\ N_2 \sim \mathcal{N}(0, 1), \tag{S1b}$$

$$Y := X + Z + N_3,\ N_3 \sim \mathcal{N}(0, 1), \tag{S1c}$$

$$W := 2Y + N_4,\ N_4 \sim \mathcal{N}(0, 1), \tag{S1d}$$

with the associated graph represented in Figure S1.

One attractive feature of this formalism is that an SCM's graphical representation entails important properties of the underlying distribution of variables $\{V_k\}$ (see e.g. (Bishop, 2006)).

PROPOSITION 1 (Markov properties). *For a given SCM $(\mathbb{S}, P_N, \mathcal{G})$, the joint distribution P_V is Markovian with respect to $\mathcal{G}$, i.e. it satisfies the following properties:*

1. *(local Markov property) each variable V_j is independent of its non-descendants given its parents $\boldsymbol{PA}_j$,*
2. *(Markov factorization property) the joint distribution $P_{\boldsymbol{V}}$ factorizes as*

$$P_{\boldsymbol{V}}(\boldsymbol{V}) = P_{\boldsymbol{V}}(V_1, \dots, V_d) = \prod_{j=1}^{d} P(V_j|\boldsymbol{PA}_j)$$

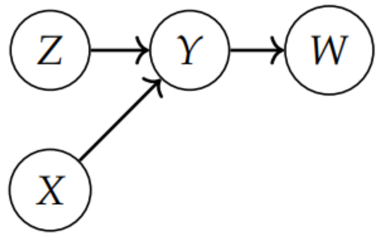

Figure S1: Graphical representation of the structural causal model of Example 1.

These two properties are rather intuitive consequences of the definition of structural equations. As an example, the graphical model represented in Figure S1 entails that $P(X,Y,Z,W) = P(W|Y)P(Y|X,Z)P(Z)P(X)$. In addition, the concept of d-separation allows assessing systematically the conditional independences between subsets of nodes in $\mathcal{G}$ based on graphical criteria (see e.g. Pearl (2000)).

DEFINITION 2 (d-separation). *A path p in graph $\mathcal{G}$ is said to be d-separated by a set of nodes Z if either: (1) p contains a chain $i \to m \to j$ or a fork $i \leftarrow m \to j$ such that the middle node m is in Z, or (2) p contains a collider $i \to m \leftarrow j$ such that the middle node m is not in Z and such than no descendant of m is in Z.*

Z is said to d-separate X from Y in $\mathcal{G}$ if and only if Z blocks every path from a node in X to a node in Y. This property is denoted $X \perp\!\!\!\perp_{\mathcal{G}} Y|Z$.

Indeed, d-separation allows stating the *global Markov property* (see e.g. (Peters et al., 2017)).

PROPOSITION 2 (Global Markov property). *For a given SCM $(\mathbb{S}, P_N, \mathcal{G})$ and subsets of nodes X, Y, Z in $\mathcal{G}$, then*

$$X \perp\!\!\!\perp_{\mathcal{G}} Y|Z \Rightarrow X \perp\!\!\!\perp_{P_V} Y|Z\,.$$

EXAMPLE 1 (continued). *The graphical model of Figure S1 implies $Z \perp\!\!\!\perp X$ and $Z \perp\!\!\!\perp W|Y$. Interestingly, it does not imply $Z \perp\!\!\!\perp X|Y$ because of the collider $Z \to Y \leftarrow X$.*

Note that d-separation only implies conditional independence. The reverse implication requires an additional assumption regarding the relation between the graph and the random variable called faithfulness (see for example (Peters et al., 2017, chapter 6)). As an example, under the faithfulness assumption, the graphical model of Figure S1 entails that X and Z are dependent given Y.

A.2 Interventions

The SCM framework allows modeling interventions performed on a system. Classical interventions in an SCM amount to impose a fixed value to a random variable, but the framework allows a much broader class of modifications of the SCM. For instance, instead of a constant, one can do (perfect) *stochastic interventions* where we impose the value of a variable to be drawn from a given distribution, independently from all exogenous variables in the SCM. More generally, taking the SCM of Definition 1, intervening on

V_k consists in replacing its structural assignment by a new one:

$$V_k := \widetilde{f}_k(\widetilde{\mathbf{PA}}_k, \widetilde{N}_k)\,.$$

The resulting modified distribution $\widetilde{P}_V = P_V^{\mathrm{do}(V_k := \widetilde{f}_k(\widetilde{\mathbf{PA}}_k, \widetilde{N}_k))}$ is called intervention distribution (see e.g. (Peters et al., 2017, chapter 6)).

EXAMPLE 1 (continued). *In the previous SCM example of Figure S1, $P_V^{do(Y:=y_0)}$ is the distribution entailed by the SCM that replaces equation (S1c) by the new structural assignment: $Y := y_0$, and keeps all other structural equations. We can see from the causal graph that the marginal distributions of X and Z will not be affected by this interventions, contrary to W.*

B PROOF OF INSENSITIVITY OF DCS TO DETERMINISTIC PERTURBATIONS

Similar to the derivation for TE in the main text, we have

$$\mathrm{DCS}(\widetilde{X}_t^2 \to \widetilde{X}_t^1) = \int \widetilde{p}(X_t^1, \boldsymbol{X}_{p,t}^1, \boldsymbol{X}_{p,t}^2) \log \frac{\widetilde{p}(X_t^1|\boldsymbol{X}_{p,t}^1, \boldsymbol{X}_{p,t}^2)}{\int \widetilde{p}(X_t^1|\boldsymbol{X}_{p,t}^1, \boldsymbol{X}_{p,t}^2)\widetilde{p}(\boldsymbol{X}_{p,t}^2)\mathrm{d}\boldsymbol{X}_{p,t}^2} \mathrm{d}X_t^1 \mathrm{d}\boldsymbol{X}_{p,t}^1 \mathrm{d}\boldsymbol{X}_{p,t}^2$$

$$= \int p(X_t^1 - U_t, \boldsymbol{X}_{p,t}^1 - \mathbf{U}_{p,t}, \boldsymbol{X}_{p,t}^2 - \mathbf{V}_{p,t}) \log \frac{p(X_t^1 - U_t|\boldsymbol{X}_{p,t}^1 - \mathbf{U}_{p,t}, \boldsymbol{X}_{p,t}^2 - \mathbf{V}_{p,t})}{\int p(X_t^1 - U_t|\boldsymbol{X}_{p,t}^1 - \mathbf{U}_{p,t}, \boldsymbol{X}_{p,t}^2 - \mathbf{V}_{p,t}) p(\boldsymbol{X}_{p,t}^2 - \mathbf{V}_{p,t})\mathrm{d}\boldsymbol{X}_{p,t}^2} \mathrm{d}X_t^1 \mathrm{d}\boldsymbol{X}_{p,t}^1 \mathrm{d}\boldsymbol{X}_{p,t}^2$$

$$= \int p(X_t^1, \boldsymbol{X}_{p,t}^1, \boldsymbol{X}_{p,t}^2) \log \frac{p(X_t^1|\boldsymbol{X}_{p,t}^1, \boldsymbol{X}_{p,t}^2)}{\int p(X_t^1|\boldsymbol{X}_{p,t}^1, \boldsymbol{X}_{p,t}^2) p(\boldsymbol{X}_{p,t}^2)\mathrm{d}\boldsymbol{X}_{p,t}^2} \mathrm{d}X_t^1 \mathrm{d}\boldsymbol{X}_{p,t}^1 \mathrm{d}\boldsymbol{X}_{p,t}^2$$

$$= \mathrm{DCS}(X_t^2 \to X_t^1)\,.$$

C SELECTION BIAS

The SCM approach allows us to draw conclusions about statistical and causal inference procedures based only on the graphical representation. One interesting question that has been addressed in such a way is selection bias (Bareinboim et al., 2014; Bareinboim and Pearl, 2012). Selection bias points to the practical issue of how selection of data points affect the inference of relevant quantities in a statistical or causal model (Hernán et al., 2004; Horwitz and Feinstein, 1978). To address this question, one can consider a binary random variable S that takes the value 1 when a data point is selected and zero otherwise. It is frequently the case that S depends (causally) on other variables of the SCM under consideration and as such it can be included as a special node in the graphical representation, as exemplified in Figure S2. Given the original distribution P over the variables, sample selection generally results in a different distribution P_S.

C.1 Recoverability with Sampling Selection Bias

In the simplest two-node SCM, the identifiability or recoverability of the effect based on different sampling methods has been investigated in (Bareinboim et al., 2014).

Figure S2A, B, C show three sampling conditions in a two-node SCM consisting of variables X and Y (with X causing Y). Sampling is represented by binary variable S in an additional node designed as

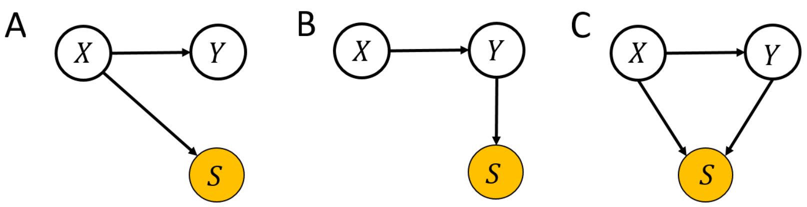

Figure S2: SCM, selection bias and recoverability (adapted from Bareinboim et al. (2014)). (A) SCM describing sample selection based on X, leading to identifiability of $P(Y|X)$ based on selected data. (B) SCM describing sample selection based on Y, leading to non-identifiability of $P(Y|X)$ based on selected data. (C) SCM describing sample selection based on both X and Y, leading to non-identifiability of $P(Y|X)$ based on selected data.

descendant for either X or Y. S takes the value 1 when a data point is selected and zero otherwise. In Figure S2A, sample selection is a function of X only, while Figure S2B describes a sample selection based on Y only. Figure S2C presents the condition where sample selection depends on both variables.

In this model, we are interested in estimating the conditional propability of $P(Y|X)$ from sampled data. What is critical is whether $P(Y|X)$ can be recovered from the joint distribution of the selected samples $(X, Y)|S = 1$ given different sampling scenarios. Bareinboim et al. (2014) show that, under standard assumptions, a necessary and sufficient condition for recoverability is conditional independence between target variable Y and selection variable S, given conditioning variable X ($Y \perp\!\!\!\perp S|X$)) such that $P(Y|X, S = 1) = P(Y|X)$. For the scenarios of Figure S2, this implies that $P(Y|X)$ can be recovered from $(X, Y)|S = 1$ in the case of Figure S2A, but not in Figure S2B and Figure S2C.

The rationale is simple according to the d-separation rules (see Section A for details). In the condition of Figure S2A, conditioning on X corresponds to the "fork" case in the d-separation rules, indicating that the conditional independence $Y \perp\!\!\!\perp S|X$ is satisfied. On the contrary, Figure S2B shows a condition where $P(Y|X)$ is not recoverable from sample selected data because the above conditional independence requirement (Y independent of S given X) is not satisfied. For Figure S2B, detailed proofs has been provided in Bareinboim et al. (2014). The case in Figure S2C corresponds to the "collider" case of d-separation where a common observed descendant induces extra dependency between the ancestors.

C.2 Recoverability with Sampling Selection Bias in VAR(p) models

Applying the aforementioned relationships between X, Y and S to VAR models, we can easily access the recoverabilities of conditionals in the SCMs, thus indicating whether the causal arrows can be estimated without further assumptions.

For example, in the SCM in Figure 3A of the main text, $\boldsymbol{X}^2_{p,t}$ d-separates S and X^1_t for every negative t', thus $P(X^1_t \mid \boldsymbol{X}^1_t, \boldsymbol{X}^2_t)$ is recoverable such that:

$$P\left(X^1_t \mid \boldsymbol{X}^1_{p,t}, \boldsymbol{X}^2_{p,t}, S\right) = P\left(X^1_t \mid \boldsymbol{X}^1_{p,t}, \boldsymbol{X}^2_{p,t}\right)$$

This is also true for positive t' as S is dependent on the indirect cause of the causal arrows. For the non-causal direction, the configuration is analogous to Figure S2C such that for negative t' there is

$$P\left(X^2_t \mid \boldsymbol{X}^1_{p,t}, \boldsymbol{X}^2_{p,t}, S\right) \neq P\left(X^2_t \mid \boldsymbol{X}^1_{p,t}, \boldsymbol{X}^2_{p,t}\right) = P\left(X^2_t \mid \boldsymbol{X}^2_{p,t}\right)$$

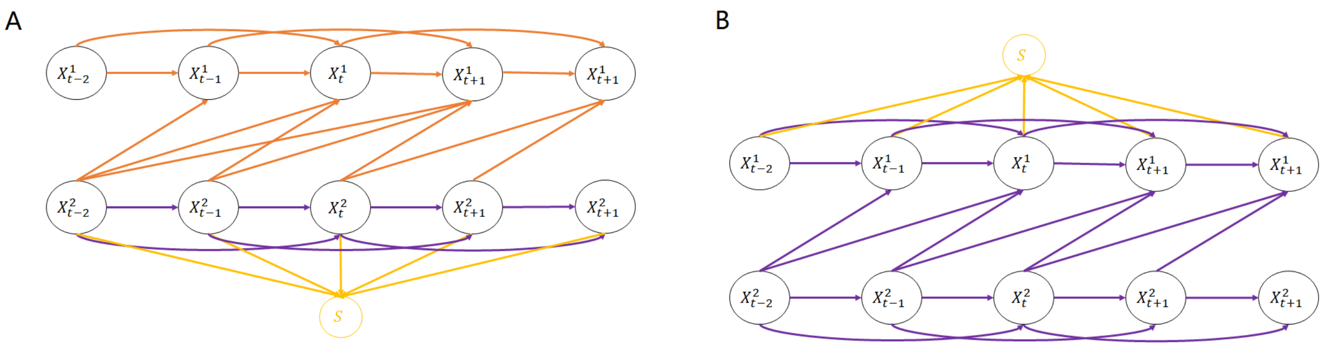

Figure S3: (A) SCM of a bi-variate VAR(2) model with uni-directional coupling from X^2 to X^1 and a selection node S depending on states of the cause variable before peri-event time ($t' <= 0$). The selection node S represents partial selection of samples due to thresholding of the detection signal obtained with template matching with the original signal. Orange indicate the recoverable arrows with the current selection node, while purple arrows indicates the unrecoverable ones. (B) The same SCM as in (A) with the selection node depending in a similar way on the effect signal.

For positive t', S only depends on the indirect cause for all arrows such that

$$P\left(X_t^2 \mid \boldsymbol{X}_{p,t}^1, \boldsymbol{X}_{p,t}^2, S\right) = P\left(X_t^2 \mid \boldsymbol{X}_{p,t}^1, \boldsymbol{X}_{p,t}^2\right) = P\left(X_t^2 \mid \boldsymbol{X}_{p,t}^2\right)$$

Interesting differences in the recoverability of $P\left(X_t^2 \mid \boldsymbol{X}_{p,t}^1, \boldsymbol{X}_{p,t}^2, S\right)$ depending on its alignment by cause or effect at different peri-event times $t' = -1$ (A), $t' = 0$ (B) and $t' = 1$ can be found in Figure S4, where $P\left(X_t^2 \mid \boldsymbol{X}_{p,t}^1, \boldsymbol{X}_{p,t}^2, S\right)$ can be recovered at peri-event time $t' = 0$ given the effect. Nonetheless, given the effect this conditional is not recoverable at all peri-event time points.

The reasoning is similar for Figure S3, which represents filtering the original signal and detecting events with template matching approaches. For Figure S3A, $P\left(X_t^1 \mid \boldsymbol{X}_{p,t}^1, \boldsymbol{X}_{p,t}^2, S\right)$ can be recovered at all peri-event time t'. For Figure 3B and Figure S3B, S can never be understood as purely dependent on the cause for negative t'. For example, for the causal arrow in $P\left(X_{t-1}^2 \mid \boldsymbol{X}_{p,t-2}^1, \boldsymbol{X}_{p,t-2}^2, S\right)$, $\boldsymbol{X}_{p,t-2}^1$ does not d-separate S and $\boldsymbol{X}_{t-1}^2$ due to the causal arrow from $\boldsymbol{X}_{t-1}^2$ to X_t^1. Thus none of the conditionals are recoverable for negative t', while this is the contrary for positive t'.

D DERIVATIONS FOR TIME-VARYING CAUSALITY MEASURES

D.1 Time-varying VAR(p) model

Following the introduction, the candidate measures include Granger causality (GC), Tranfer Entropy (TE) and Causal Strength (CS) (Janzing et al., 2013), where we extend them into a time-varying version to address the non-stationarity of transient events. In addition to their potential information theoretic formulations, the measures will also be formulated in a time-varying bivariate system consisting of two variables X^1 and X^2. The system is assumed to be a inhomogeneous bivariate p-ordered Vector Autoregressive (VAR(p)) model. At each time t, the current state $\boldsymbol{X}_t = [X_t^1, X_t^2]^\top$ is a linear function of the past (lagged) p states gathered in the vector

$$\boldsymbol{X}_{p,t} = [{\boldsymbol{X}_{p,t}^1}^\top, {\boldsymbol{X}_{p,t}^2}^\top]^\top = [X_{t-1}^1, X_{t-2}^1, ..., X_{t-p}^1, X_{t-1}^2, X_{t-2}^2, ..., X_{t-p}^2]^\top \tag{S2}$$

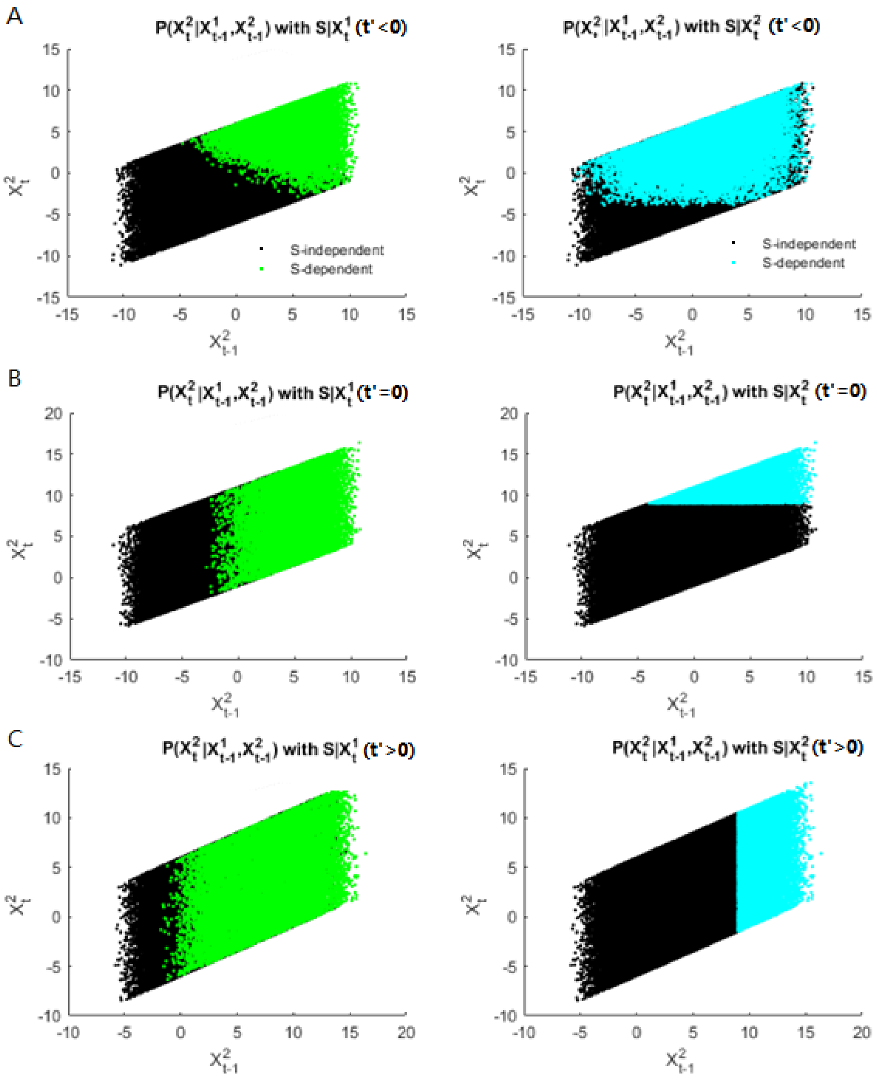

Figure S4: Illustration of recoverability of the non-causal direction at different peri-event time. Joint distributions of the lagged variables and the cause variable of a VAR(1) model with uniformly distributed innovations (same as in Figure 3E) at peri-event time $t' = -1$ (A), $t' = 0$ (B) and $t' = 1$ (C). Black represents ground truth alignments while green and cyan marks the distributions with effect alignment (left) and cause alignment (right). Apparently, before the detection point (i.e. $t' < 0$) the conditional is not recoverable for both alignments but after the detection point both are recoverable. At $t' = 0$ it is only recoverable for effect alignment.

and the exogeneous inputs as the innovation term

$$\boldsymbol{\eta}_t = [\eta_t^1, \eta_t^2]^\top$$

in the following form

$$\boldsymbol{X}_t := A_t \boldsymbol{X}_{p,t} + \boldsymbol{\eta}_t \, , \boldsymbol{\eta}_t \sim \mathcal{N}(\mathbf{k}_t, \Sigma_t) \, , \quad \text{(S3)}$$

where the autoregressive coefficient $A_t = \begin{bmatrix} \mathbf{a}_t^\top & \mathbf{b}_t^\top \\ \mathbf{c}_t^\top & \mathbf{d}_t^\top \end{bmatrix}$, the innovation mean $\mathbf{k}_t = \begin{bmatrix} k_t^1 \\ k_t^2 \end{bmatrix}$, and innovation covariance $\Sigma_t = \begin{bmatrix} \sigma_{1,t}^2 & 0 \\ 0 & \sigma_{2,t}^2 \end{bmatrix}$ are the model parameters. The "colon equals" sign suggests that the VAR model can be interpreted in the SCM framework (Section A). Elements of the coefficient matrix $\mathbf{a}_t$,$\mathbf{b}_t$,$\mathbf{c}_t$ and $\mathbf{d}_t$ are all p-dimensional vectors. Expanding the vector version of Eq. S3 using Eq. S2, the equation can be rewritten as:

$$X_t^1 = \mathbf{a}_t^\top \boldsymbol{X}_{p,t}^1 + \mathbf{b}_t^\top \boldsymbol{X}_{p,t}^2 + \eta_t^1 \,, \quad \eta_t^1 \sim \mathcal{N}(k_t^1,\, \sigma_{1,t}^2)\,, \tag{S4}$$

$$X_t^2 = \mathbf{c}_t^\top \boldsymbol{X}_{p,t}^1 + \mathbf{d}_t^\top \boldsymbol{X}_{p,t}^2 + \eta_t^2 \,, \quad \eta_t^2 \sim \mathcal{N}(k_t^2,\, \sigma_{2,t}^2)\,. \tag{S5}$$

D.2 Derivation of KL-divergence between two uni-variate Gaussians

Time-varying TE, DCS and rDCS are formulated as the KL divergence between the corresponding actual and counterfactual conditions. Thus we first present the KL divergence between two uni-variate Gaussian variables.

We denote the Gaussian for the actual condition as $p(x) = \mathcal{N}(\mu_a, \sigma_a^2)$, and the counterfactual gaussian as $q(x) = \mathcal{N}(\mu_c, \sigma_c^2)$. Then the KL divergence between $p(x)$ and $q(x)$ is

$$\begin{aligned}
D_{KL}(p||q) &= -\int p(x)\log q(x)dx + \int p(x)\log p(x)dx = \int \left[\log(p(x)) - log(q(x))\right] p(x)dx \\
&= \int \left[-\frac{1}{2}\log(2\pi) - \log(\sigma_a) - \frac{1}{2}\left(\frac{x-\mu_a}{\sigma_a}\right)^2 + \frac{1}{2}\log(2\pi) + \log(\sigma_c) + \frac{1}{2}\left(\frac{x-\mu_c}{\sigma_c}\right)^2\right] \times \\
&\qquad \frac{1}{\sqrt{2\pi}\sigma_a}\exp\left[-\frac{1}{2}\left(\frac{x-\mu_a}{\sigma_a}\right)^2\right] dx \\
&= \int \left\{\log\frac{\sigma_c}{\sigma_a} + \frac{1}{2}\left[\left(\frac{x-\mu_c}{\sigma_c}\right)^2 - \left(\frac{x-\mu_a}{\sigma_a}\right)^2\right]\right\} \times \frac{1}{\sqrt{2\pi}\sigma_a}\exp\left[-\frac{1}{2}\left(\frac{x-\mu_a}{\sigma_a}\right)^2\right] dx \\
&= \mathbb{E}_p\left[\log\frac{\sigma_c}{\sigma_a} + \frac{1}{2}\left[\left(\frac{x-\mu_c}{\sigma_c}\right)^2 - \left(\frac{x-\mu_a}{\sigma_a}\right)^2\right]\right] \\
&= \log\frac{\sigma_c}{\sigma_a} + \frac{1}{2\sigma_c^2}\mathbb{E}_p\left[(X-\mu_c)^2\right] - \frac{1}{2\sigma_a^2}\mathbb{E}_p\left[(X-\mu_a)^2\right] \\
&= \log\frac{\sigma_c}{\sigma_a} + \frac{1}{2\sigma_c^2}\mathbb{E}_p\left[(X-\mu_c)^2\right] - \frac{1}{2}
\end{aligned}$$

Note that

$$\begin{aligned}
(X-\mu_c)^2 &= (X-\mu_a+\mu_a-\mu_c)^2 \\
&= (X-\mu_a)^2 + 2(X-\mu_a)(\mu_a-\mu_c) + (\mu_a-\mu_c)^2\,.
\end{aligned}$$

Therefore,

$$D_{KL}(\mathcal{N}(\mu_a,\,\sigma_a^2)||\mathcal{N}(\mu_c,\,\sigma_c^2)) = \log\frac{\sigma_c}{\sigma_a} + \frac{1}{2\sigma_c^2}\mathbb{E}_p\left[(X-\mu_a)^2\right]$$
$$+\,2(\mu_a-\mu_c)\mathbb{E}_p\left[X-\mu_a\right] + (\mu_a-\mu_c)^2 - \frac{1}{2}$$
$$= \frac{1}{2}\log\frac{\sigma_c^2}{\sigma_a^2} + \frac{\sigma_a^2+(\mu_a-\mu_c)^2}{2\sigma_c^2} - \frac{1}{2} \quad \text{(S6)}$$

D.3 Conditional mean and variance for the actual condition

The actual condition defined for TE, DCS and rDCS is that the current state of X_t^1 is dependent on the past of both X^1 and X^2, denoted as

$$\mathcal{N}(\mu_a,\sigma_a^2) = p(X_t^1|\boldsymbol{X}_{p,t}^1,\boldsymbol{X}_{p,t}^2)\,.$$

The dynamics of X_t^1 is described by the structural equation

$$X_t^1 = \mathbf{a}_t^\top\boldsymbol{X}_{p,t}^1 + \mathbf{b}_t^\top\boldsymbol{X}_{p,t}^2 + \eta_t^1\,, \quad \eta_t^1 \sim \mathcal{N}(k_t^1,\,\sigma_{1,t}^2)\,. \quad \text{(S7)}$$

Therefore, the time-varying conditional mean and variance can be derived as

$$\mu_a = \mathbb{E}[\mathbf{a}_t^\top\boldsymbol{X}_{p,t}^1 + \mathbf{b}_t^\top\boldsymbol{X}_{p,t}^2 + \eta_t^1|\boldsymbol{X}_{p,t}^1,\boldsymbol{X}_{p,t}^2] = \mathbf{a}_t^\top\boldsymbol{X}_{p,t}^1 + \mathbf{b}_t^\top\boldsymbol{X}_{p,t}^2 + k_t^1$$
$$\sigma_a^2 = \mathrm{Var}[\mathbf{a}^\top\boldsymbol{X}_{p,t}^1 + \mathbf{b}^\top\boldsymbol{X}_{p,t}^2 + \eta_t^1|\boldsymbol{X}_{p,t}^1,\boldsymbol{X}_{p,t}^2] = \mathrm{Var}[\eta_t^1|\boldsymbol{X}_{p,t}^1,\boldsymbol{X}_{p,t}^2] = \sigma_{1,t}^2$$

D.4 Transfer Entropy

For TE, as the conditional probability representing the post-intervention world takes the form

$$\mathcal{N}(\mu_c,\sigma_c^2) = p(X_t^1|\boldsymbol{X}_{p,t}^1)\,.$$

Resulting from the same model in Eq. S7, the mean and variance can be derived as

$$\mu_c = \mathbb{E}[\mathbf{a}_t^\top\boldsymbol{X}_{p,t}^1 + \mathbf{b}_t^\top\boldsymbol{X}_{p,t}^2 + \eta_t^1|\boldsymbol{X}_{p,t}^1] = \mathbf{a}_t^\top\boldsymbol{X}_{p,t}^1|\boldsymbol{X}_{p,t}^1 + \mathbf{b}_t^\top\mathbb{E}[\boldsymbol{X}_{p,t}^2|\boldsymbol{X}_{p,t}^1] + k_t^1$$
$$\sigma_c^2 = \mathrm{Var}[\mathbf{a}^\top\boldsymbol{X}_{p,t}^1 + \mathbf{b}^\top\boldsymbol{X}_{p,t}^2 + \eta_t^1|\boldsymbol{X}_{p,t}^1] = \mathbf{b}_t^\top\mathrm{Cov}[\boldsymbol{X}_{p,t}^2|\boldsymbol{X}_{p,t}^1]\mathbf{b}_t + \sigma_{1,t}^2$$

while

$$\mathbb{E}_{(\boldsymbol{X}_{p,t}^1,\boldsymbol{X}_{p,t}^2)}[(\mu_a-\mu_c)^2] = \mathbb{E}_{(\boldsymbol{X}_{p,t}^1,\boldsymbol{X}_{p,t}^2)}[(\mathbf{b}_t^\top(\boldsymbol{X}_{p,t}^2 - \mathbb{E}[\boldsymbol{X}_{p,t}^2|\boldsymbol{X}_{p,t}^1]))^2]$$
$$= \mathbf{b}_t^\top\mathbb{E}[(\boldsymbol{X}_{p,t}^2 - \mathbb{E}|\boldsymbol{X}_{p,t}^2])(\boldsymbol{X}_{p,t}^2 - \mathbb{E}|\boldsymbol{X}_{p,t}^2|\boldsymbol{X}_{p,t}^1])^\top]\mathbf{b}_t = \mathbf{b}_t^\top\,\mathrm{Cov}[\boldsymbol{X}_{p,t}^2|\boldsymbol{X}_{p,t}^1]]\mathbf{b}_t$$

Plugging the expressions of μ_a, μ_c, σ_a^2 and σ_c^2 into Eq. S6, the KL divergence can be derived as

$$\mathrm{TE}(X_t^2 \to X_t^1) = D_{KL}(\mathcal{N}(\mu_a,\,\sigma_a^2)||\mathcal{N}(\mu_c,\,\sigma_c^2)) = \frac{1}{2}\log\frac{\mathbf{b}_t^\top\mathrm{Cov}[\boldsymbol{X}_{p,t}^2|\boldsymbol{X}_{p,t}^1]\mathbf{b}_t + \sigma_{1,t}^2}{\sigma_{1,t}^2}$$

As $\boldsymbol{X}^1_{p,t}$ and $\boldsymbol{X}^2_{p,t}$ are jointly Gaussian, the conditional variance takes the form

$$\mathrm{Cov}[\boldsymbol{X}^2_{p,t}|\boldsymbol{X}^1_{p,t}] = \Sigma_{\boldsymbol{X}^2_p} - \Sigma_{\boldsymbol{X}^1_p\boldsymbol{X}^2_p}\Sigma^{-1}_{\boldsymbol{X}^1_p}\Sigma_{\boldsymbol{X}^2_p\boldsymbol{X}^1_p}\,.$$

Therefore, the expression of time-varying TE should be

$$\mathrm{TE}(X^2_t \to X^1_t) = \frac{1}{2}\log\frac{\sigma^2_{1,t} + \mathbf{b}_t^\top\Sigma_{\boldsymbol{X}^2_p}\mathbf{b}_t - \mathbf{b}_t^\top\Sigma_{\boldsymbol{X}^1_p\boldsymbol{X}^2_p}\Sigma^{-1}_{\boldsymbol{X}^1_p}\Sigma_{\boldsymbol{X}^2_p\boldsymbol{X}^1_p}\mathbf{b}_t}{\sigma^2_{1,t}}\,. \tag{S8}$$

D.5 Dynamic Causal Strength

For DCS, the post-intervention conditional is

$$\mathcal{N}(\mu_c, \sigma_c^2) = p^{do(X^1_t := f(\boldsymbol{X}^1_{p,t}, \boldsymbol{X}^{2\,\prime}_{p,t}, \eta^1_t))}(X^1_t|\boldsymbol{X}^1_{p,t}, \boldsymbol{X}^2_{p,t})$$

with $X^1_t = \mathbf{a}_t^\top\boldsymbol{X}^1_{p,t} + \mathbf{b}_t^\top\boldsymbol{X}^{2\,\prime}_{p,t} + \eta^1_t$, and $\boldsymbol{X}^{2\,\prime}_{p,t}$ is a random sample drawn from the distribution $p(\boldsymbol{X}^2_{p,t})$. Then the conditional mean is:

$$\mu_c = \mathbb{E}[\mathbf{a}_t^\top\boldsymbol{X}^1_{p,t} + \mathbf{b}_t^\top\boldsymbol{X}^{2\,\prime}_{p,t} + \eta^1_t|\boldsymbol{X}^1_{p,t}, \boldsymbol{X}^2_{p,t}] = \mathbf{a}_t^\top\boldsymbol{X}^1_{p,t} + \mathbf{b}_t^\top\mathbb{E}[\boldsymbol{X}^2_{p,t}] + \eta^1_t\,.$$

The conditional variance is:

$$\begin{aligned}\sigma_a^2 &= \mathrm{Var}[\mathbf{a}^\top\boldsymbol{X}^1_{p,t} + \mathbf{b}^\top\boldsymbol{X}^2_{p,t} + \eta^1_t|\boldsymbol{X}^1_{p,t}, \boldsymbol{X}^2_{p,t}] \\ &= \mathrm{Var}[\mathbf{b}^\top\boldsymbol{X}^{2\,\prime}_{p,t}|\boldsymbol{X}^2_{p,t}] + \mathrm{Var}[\eta^1_t] = \mathbf{b}^\top\,\mathrm{Cov}[\boldsymbol{X}^2_{p,t}]\mathbf{b} + \sigma^2_{1,t}\,,\end{aligned}$$

with $\mathrm{Cov}[\boldsymbol{X}^2_{p,t}] = \mathbb{E}[(\boldsymbol{X}^2_{p,t} - \mathbb{E}|\boldsymbol{X}^2_{p,t}])(\boldsymbol{X}^2_{p,t} - \mathbb{E}|\boldsymbol{X}^2_{p,t}])^\top]$. This leads to DCS defined as the KL divergence between the actual and post-intervention worlds:

$$\begin{aligned}\mathrm{DCS}(X^2_t \to X^1_t) &= \mathbb{E}_{(\boldsymbol{X}^1_{p,t},\boldsymbol{X}^2_{p,t})}\left[\frac{1}{2}\log\frac{\sigma_c^2}{\sigma_a^2} - \frac{1}{2} + \frac{1}{2}\cdot\frac{\sigma_a^2 + (\mu_a - \mu_c)^2}{\sigma_c^2}\right] \\ &= \frac{1}{2}\log\frac{\sigma_c^2}{\sigma_a^2} - \frac{1}{2} + \frac{1}{2}\cdot\frac{\sigma_a^2 + \mathbb{E}_{(\boldsymbol{X}^1_{p,t},\boldsymbol{X}^2_{p,t})}[(\mu_a - \mu_c)^2]}{\sigma_c^2}\,,\end{aligned}$$

with

$$\begin{aligned}\mathbb{E}_{(\boldsymbol{X}^1_{p,t},\boldsymbol{X}^2_{p,t})}[(\mu_a - \mu_c)^2] &= \mathbb{E}_{(\boldsymbol{X}^1_{p,t},\boldsymbol{X}^2_{p,t})}[(\mathbf{b}_t^\top(\boldsymbol{X}^2_{p,t} - \mathbb{E}[\boldsymbol{X}^2_{p,t}]))^2] \\ &= \mathbf{b}_t^\top\mathbb{E}[(\boldsymbol{X}^2_{p,t} - \mathbb{E}|\boldsymbol{X}^2_{p,t}])(\boldsymbol{X}^2_{p,t} - \mathbb{E}|\boldsymbol{X}^2_{p,t}])^\top]\mathbf{b}_t = \mathbf{b}_t^\top\,\mathrm{Cov}[\boldsymbol{X}^2_{p,t}]\mathbf{b}_t\,.\end{aligned}$$

Therefore the expression of DCS can be obtained by plugging-in the means and variances of the two Gaussian distributions,

$$\mathrm{DCS}(X_t^2 \to X_t^1) = \frac{1}{2}\log\frac{\mathbf{b}_t^\top \operatorname{Cov}[\boldsymbol{X}_{p,t}^2]\mathbf{b}_t + \sigma_{1,t}^2}{\sigma_{1,t}^2} - \frac{1}{2} + \frac{1}{2}\cdot\frac{\sigma_{1,t}^2 + \mathbf{b}_t^\top \operatorname{Cov}[\boldsymbol{X}_{p,t}^2]\mathbf{b}_t}{\mathbf{b}_t^\top \operatorname{Cov}[\boldsymbol{X}_{p,t}^2]\mathbf{b}_t + \sigma_{1,t}^2} = \frac{1}{2}\log\frac{\mathbf{b}_t^\top \operatorname{Cov}[\boldsymbol{X}_{p,t}^2]\mathbf{b}_t + \sigma_{1,t}^2}{\sigma_{1,t}^2} \tag{S9}$$

D.6 Relative Dynamic Causal Strength

The relative Causal Strength is 'relative' in the sense that the intervention involves an independent copy of a baseline state $\boldsymbol{X}_{p,t_{ref}}^2$, instead of the lagged state $\boldsymbol{X}_{p,t}^{2\;\prime}$, such that $X_t^{1\prime} = \mathbf{a}_t^\top \boldsymbol{X}_{p,t}^1 + \mathbf{b}_t^\top \boldsymbol{X}_{p,t_{ref}}^2 + \eta_t^1$ for the post-intervention world. This leads to the conditional

$$\mathcal{N}(\mu_c, \sigma_c^2) = p^{do(X_t^1 := f(\boldsymbol{X}_{p,t}^1, \boldsymbol{X}_{p,t_{ref}}^2, \eta_t^1))}(X_t^1 | \boldsymbol{X}_{p,t}^1, \boldsymbol{X}_{p,t}^2)\,.$$

where the conditional mean and variance expressions are:

$$\mu_c = \mathbb{E}[\mathbf{a}_t^\top \boldsymbol{X}_{p,t}^1 + \mathbf{b}_t^\top \boldsymbol{X}_{p,t_{ref}}^2 + \eta_t^1 | \boldsymbol{X}_{p,t}^1, \boldsymbol{X}_{p,t}^2] = \mathbf{a}_t^\top \boldsymbol{X}_{p,t}^1 + \mathbf{b}_t^\top \mathbb{E}[\boldsymbol{X}_{p,t_{ref}}^2] + \eta_t^1$$

and

$$\begin{aligned}\sigma_c^2 &= \operatorname{Var}[\mathbf{a}_t^\top \boldsymbol{X}_{p,t}^1 + \mathbf{b}_t^\top \boldsymbol{X}_{p,t_{ref}}^2 + \eta_t^1 | \boldsymbol{X}_{p,t}^1, \boldsymbol{X}_{p,t}^2] \\ &= \operatorname{Var}[\mathbf{a}_t^\top \boldsymbol{X}_{p,t_{ref}}^2 | \boldsymbol{X}_{p,t}^2] + \operatorname{Var}[\eta_t^1] = \mathbf{b}_t^\top \operatorname{Cov}[\boldsymbol{X}_{p,t_{ref}}^2]\mathbf{b}_t + \sigma_{1,t}^2\,,\end{aligned}$$

with $\operatorname{Cov}[\boldsymbol{X}_{p,t_{ref}}^2] = \mathbb{E}[(\boldsymbol{X}_{p,t_{ref}}^2 - \mathbb{E}[\boldsymbol{X}_{p,t_{ref}}^2])(\boldsymbol{X}_{p,t_{ref}}^2 - \mathbb{E}[\boldsymbol{X}_{p,t_{ref}}^2])^\top]$.

The Gaussian KL divergence expression of rDCS has the term

$$\begin{aligned}\mathbb{E}_{(\boldsymbol{X}_{p,t}^1, \boldsymbol{X}_{p,t}^2)}[(\mu_a - \mu_c)^2] &= \mathbb{E}_{(\boldsymbol{X}_{p,t}^1, \boldsymbol{X}_{p,t}^2)}[(\mathbf{b}_t^\top (\boldsymbol{X}_{p,t}^2 - \mathbb{E}[\boldsymbol{X}_{p,t_{ref}}^2]))^2] \\ &= \mathbf{b}_t^\top \mathbb{E}[(\boldsymbol{X}_{p,t}^2 - \mathbb{E}[\boldsymbol{X}_{p,t_{ref}}^2])(\boldsymbol{X}_{p,t}^2 - \mathbb{E}[\boldsymbol{X}_{p,t_{ref}}^2])^\top]\mathbf{b}_t\,.\end{aligned}$$

Therefore, the rDCS expression for a Gaussian SVAR is

$$\mathrm{rDCS}(X_t^2 \to X_t^1) = \frac{1}{2}\log\frac{\sigma_{1,t}^2 + \mathbf{b}_t^\top \operatorname{Cov}[\boldsymbol{X}_{p,t_{ref}}^2]\mathbf{b}_t}{\sigma_{1,t}^2} - \frac{1}{2} + \frac{1}{2}\cdot\frac{\sigma_{1,t}^2 + \mathbf{b}_t^\top \mathbb{E}[(\boldsymbol{X}_{p,t}^2 - \mathbb{E}[\boldsymbol{X}_{p,t_{ref}}^2])(\boldsymbol{X}_{p,t}^2 - \mathbb{E}[\boldsymbol{X}_{p,t_{ref}}^2])^\top]\mathbf{b}_t}{\sigma_{1,t}^2 + \mathbf{b}_t^\top \operatorname{Cov}[\boldsymbol{X}_{p,t_{ref}}^2]\mathbf{b}_t}\,. \tag{S10}$$

E DYNAMICAL SYSTEM AS A SVAR(2) MODEL FOR NUMERICAL SIMULATION

The pair of second order Ordinary Differential Equations (ODEs) with noise in Section 3.1 (Equation 13) can be rewritten as four first order ODEs:

$$\frac{dx}{dt} = u(t)\,, \tag{S11a}$$

$$\frac{du}{dt} = au(t) + bx(t) + 0 \cdot y(t) + n_x(t)\,, \tag{S11b}$$

$$\frac{dy}{dt} = z(t)\,, \tag{S11c}$$

$$\frac{dz}{dt} = mz(t) + ny(t) + cx(t) + n_y(t)\,. \tag{S11d}$$

where $a = -2\zeta_x\omega_x$, $b = -\omega_x^2$, $m = -2\zeta_y\omega_y$, $n = -\omega_y^2$. Then they can be numerically integrated via either Euler method or the 4-th order Runge-Kutta method. We show here how these models can be understood and modeled by a linear 2-nd order Structural Vector Autoregressive model (SVAR(2)).

E.1 Euler's method

The Euler's 1-step algorithm shows that with a time step h Equation S11 can be approximated by:

$$x(t+1) = x(t) + hu(t)\,, \tag{S12a}$$

$$u(t+1) = u(t) + h(au(t) + bx(t) + 0 \cdot y(t) + n_x(t))\,, \tag{S12b}$$

$$y(t+1) = y(t) + hz(t)\,, \tag{S12c}$$

$$z(t+1) = z(t) + h(mz(t) + by(t) + cx(t) + n_x(t))\,. \tag{S12d}$$

From Equation S12a, we can obtain the same relationship at the next time step

$$x(t+2) = x(t+1) + hu(t+1)\,. \tag{S13}$$

Combining Equation S12a, S12b and S13, one can eliminate the variable u, yielding

$$x(t+2) = (2+ah)x(t+1) + (-1-ah+bh^2)x(t) + 0 \cdot y(t) + n_x(t)\,. \tag{S14}$$

Similarly, combining Equation S12c and S12d, it is possible to obtain the 2-order recursion for the variable y:

$$y(t+2) = (2+mh)y(t+1) + (-1-mh+nh^2)y(t) + cx(t) + n_y(t)\,. \tag{S15}$$

Together, Equation S14 and S15 can be interpreted as an SVAR(2) model.

E.2 Runge-Kutta method

We take the last two equations for example, which is a more complicated example compared to the first two equations due to the coupling term. Notably, the two groups of equations can be separated as the coupling term can be understood as within the innovation, thus not interfering with the recursive numerical

integration. Equation S15a and S15b can be rewritten in the vector form:

$$\frac{d}{dt}\begin{bmatrix}u(t)\\x(t)\end{bmatrix} = \begin{bmatrix}a & b\\1 & 0\end{bmatrix}\begin{bmatrix}u(t)\\x(t)\end{bmatrix} + \begin{bmatrix}0 \cdot y(t) + n_x(t)\\cx(t) + n_y(t)\end{bmatrix}. \tag{S16}$$

Equation S16 can be written in a compact form if we define $\mathbf{x}(t) = \begin{bmatrix}u(t)\\x(t)\end{bmatrix}, H = \begin{bmatrix}a & b\\1 & 0\end{bmatrix}, \mathbf{n} = \begin{bmatrix}0 \cdot y(t) + n_x(t)\\0\end{bmatrix}$, such that

$$\frac{d\mathbf{x}}{dt}(t) = H\mathbf{x}(t) + \mathbf{n}(t). \tag{S17}$$

Then the vector-version Runge-Kutta can be applied to the Equation S17, such that the future state of $\mathbf{x}$ can be calculated from the current state:

$$\mathbf{x}(t+1) = \mathbf{x}(t) + \frac{h}{6}(\mathbf{a} + 2\mathbf{b} + 2\mathbf{c} + \mathbf{d}), \tag{S18}$$

where

$$\mathbf{a} = H(\mathbf{x}(t)) + \mathbf{n}(t)\,, \tag{S19a}$$

$$\mathbf{b} = H(\mathbf{x}(t) + \frac{h}{2}\mathbf{a}) + \mathbf{n}(t)\,, \tag{S19b}$$

$$\mathbf{c} = H(\mathbf{x}(t) + \frac{h}{2}\mathbf{b}) + \mathbf{n}(t)\,, \tag{S19c}$$

$$\mathbf{d} = H(\mathbf{x}(t) + \mathbf{c}) + \mathbf{n}(t)\,. \tag{S19d}$$

By unfolding each sub-step in Equation S19 and plugging them into Equation S18, it is easy to find a linear relationship between $\mathbf{x}(t+1)$, $\mathbf{x}(t)$ and $\mathbf{n}(t)$. We introduced some new parameters to describe this relationship:

$$\mathbf{x}(t+1) = W\mathbf{x}(t) + V\mathbf{n}(t), \tag{S20}$$

with

$$W = \begin{bmatrix}w_{11} & w_{12}\\w_{21} & w_{22}\end{bmatrix}, V = \begin{bmatrix}v_1\\v_2\end{bmatrix}, \mathbf{n} = \begin{bmatrix}n_1\\n_2\end{bmatrix}.$$

Notably, $n_1 = 0 \cdot y(t) + n_x(t)$, $n_2 = 0$. Rewriting Equation S20 in a element-wise form, one gets

$$u(t+1) = w_{11}u(t) + w_{12}x(t) + v_1 n_x(t) \tag{S21a}$$

$$x(t+1) = w_{21}u(t) + w_{22}x(t). \tag{S21b}$$

Similar to the Euler's method, we can obtain the recursion for the next step as:

$$x(t+2) = w_{21}u(t+1) + w_{22}x(t+1)\,. \tag{S22}$$

Combining Equation S22 with Equation S21a, we can obtain the a second-order difference equation for the variable x:

$$x(t+2) = (w_{11} + w_{22})x(t+1) + (w_{21}w_{12} - w_{11}w_{22})x(t) + w_{21}v_1 n_x(t)\,. \tag{S23}$$

Similarly, we can obtain a linear second order difference equation for y, which in together forms a bi-variate VAR(2) model.

F ADDITIONAL EXPERIMENT FOR PUTATIVE EFFECT ALIGNMENT

In the simulated VAR(4) model for the experiment of deterministic perturbations in Section 3.2, due to the large perturbation and strong coupling, the error caused by putative effect alignment is small. For a better illustration of this point, we conducted an additional experiment with the same VAR(4) model but weaker perturbation (40%) and smaller coupling strength (10%). The results are shown in Supplementary Figure S5, S6 and S7.

While Supplementary Figure S6 is consistent with Figure 5 for putative cause alignment (see a zoomed version of Figure 5D in Figure S8), Figure S5C shows a transient bias of VAR coefficient estimation at $t' = 0$ with putative effect alignment, which contributes to the sharp decrease in estimation of TE, DCS and rDCS at $t' = 0$ for both single-time and smoothed case. Compared to the ground truth where the transient rDCS in the causal direction is never smaller than the baseline rDCS, rDCS at $t' = 0$ is close to zero, which is against the fact that the two nodes are constantly coupled. This may reflect the selection bias of causal influence estimation using event detection based on putative effects.

In this uni-directional example we do not observe a wrong estimation of the causal direction by putative effect alignment. However, we also checked the case where the events are aligned by the ground truth effect (Figure S7B), where the dominant direction of causation does not match ground truth, consistently with what we argued in the main text (Section 2.5, second paragraph): inappropriate alignment may cause wrong inference of the direction of causation.

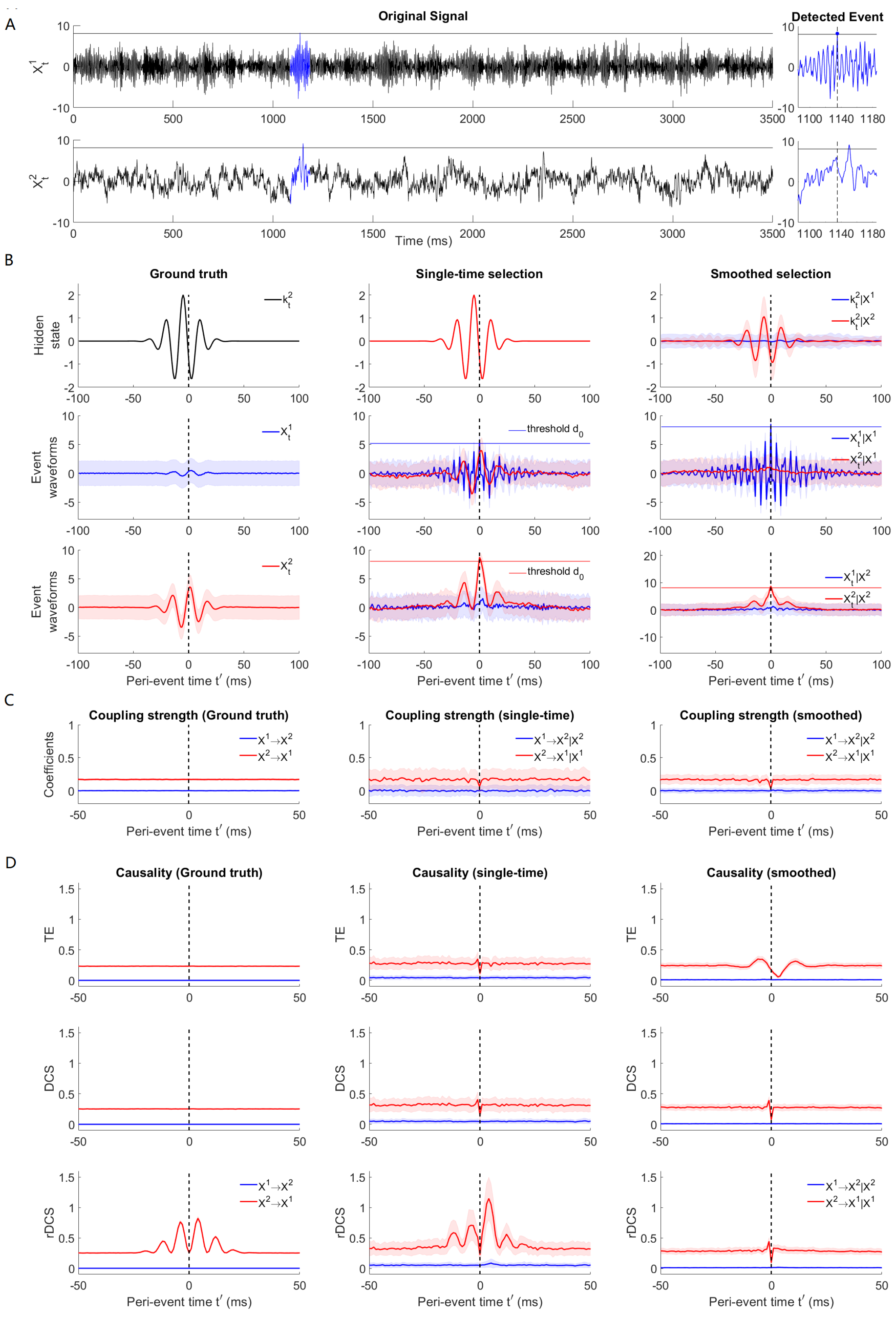

Figure S5: Causal analysis for simulated perturbation events aligned by putative effects. (A) Example signal traces of the bi-variate VAR(4) system with weaker uni-directional coupling strength (black). Blue traces mark one example events detected by thresholding over the effect X_t^1. The blue dot shows the reference point. (B) (Top) Hidden states for ground-truth alignment (left), single time selection of the ground truth event ensembles due to thresholding (middle) and events aligned by local peaks over threshold (right). (Middle) ground truth event ensemble for X_t^1 (left) and bi-variate ensembles of the other two selections aligned by X_t^1 (middle, right). Thin blue line represents the threshold in X_t^1. (Bottom) Same

Figure S5: **(continued)** settings as in (middle) but aligned by X_t^2. (C) Example elements of coupling strength in the ground truth directions $X_t^2 \to X_t^1$ (red) and the opposite direction $X_t^1 \to X_t^2$ (blue) for 3 types of event ensembles aligned by putative effect. (D) TE (left), DCS (middle) and rDCS (right) for all 3 types of event ensembles aligned by putative effect.

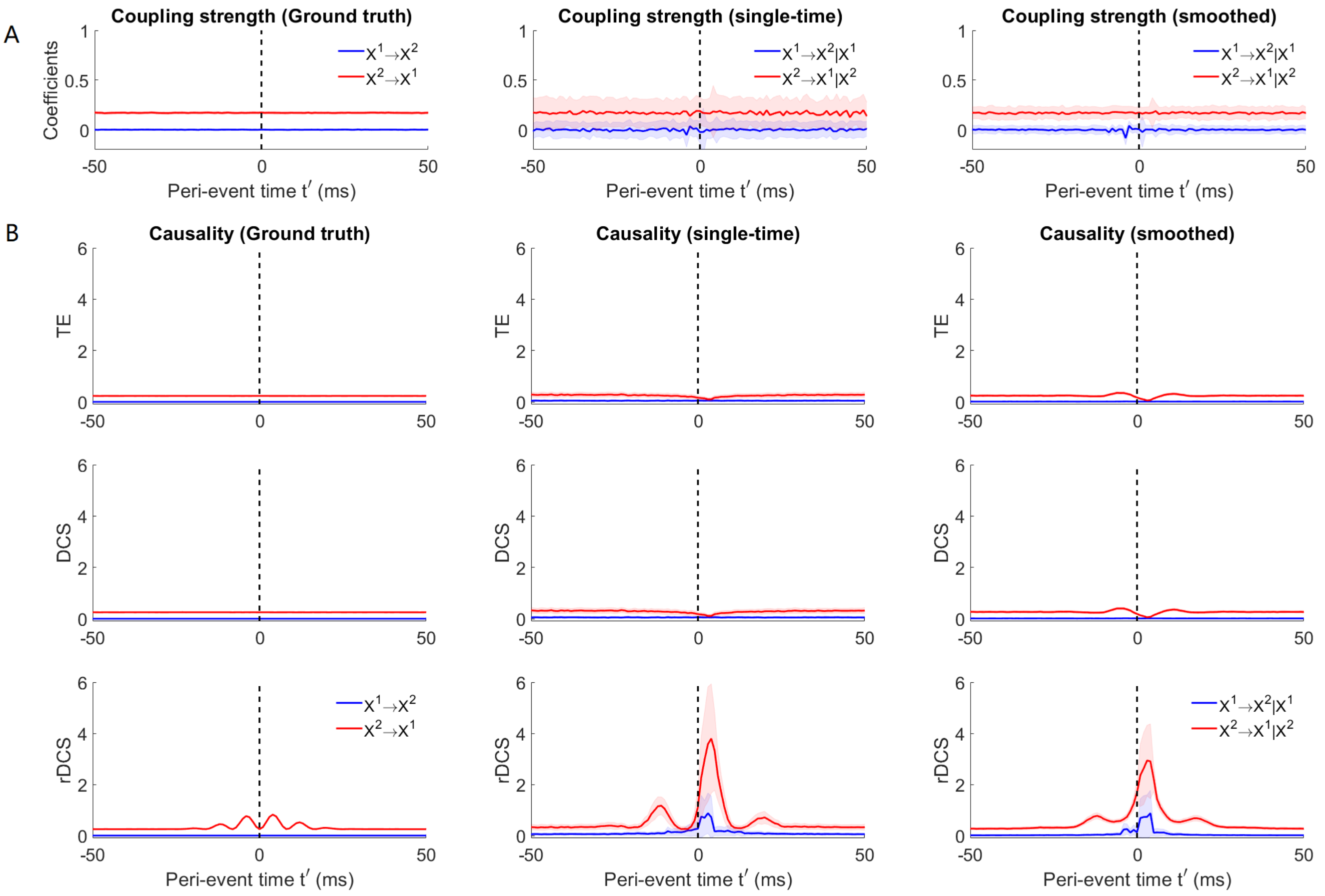

Figure S6: Causal analysis for simulated perturbation events with weaker coupling aligned by putative causes. (A) Example elements of coupling strength in the ground truth directions $X_t^2 \to X_t^1$ (red) and the opposite direction $X_t^1 \to X_t^2$ (blue) for 3 types of event ensembles aligned by putative cause. (B) TE (left), DCS (middle) and rDCS (right) for all 3 types of event ensembles aligned by putative cause.

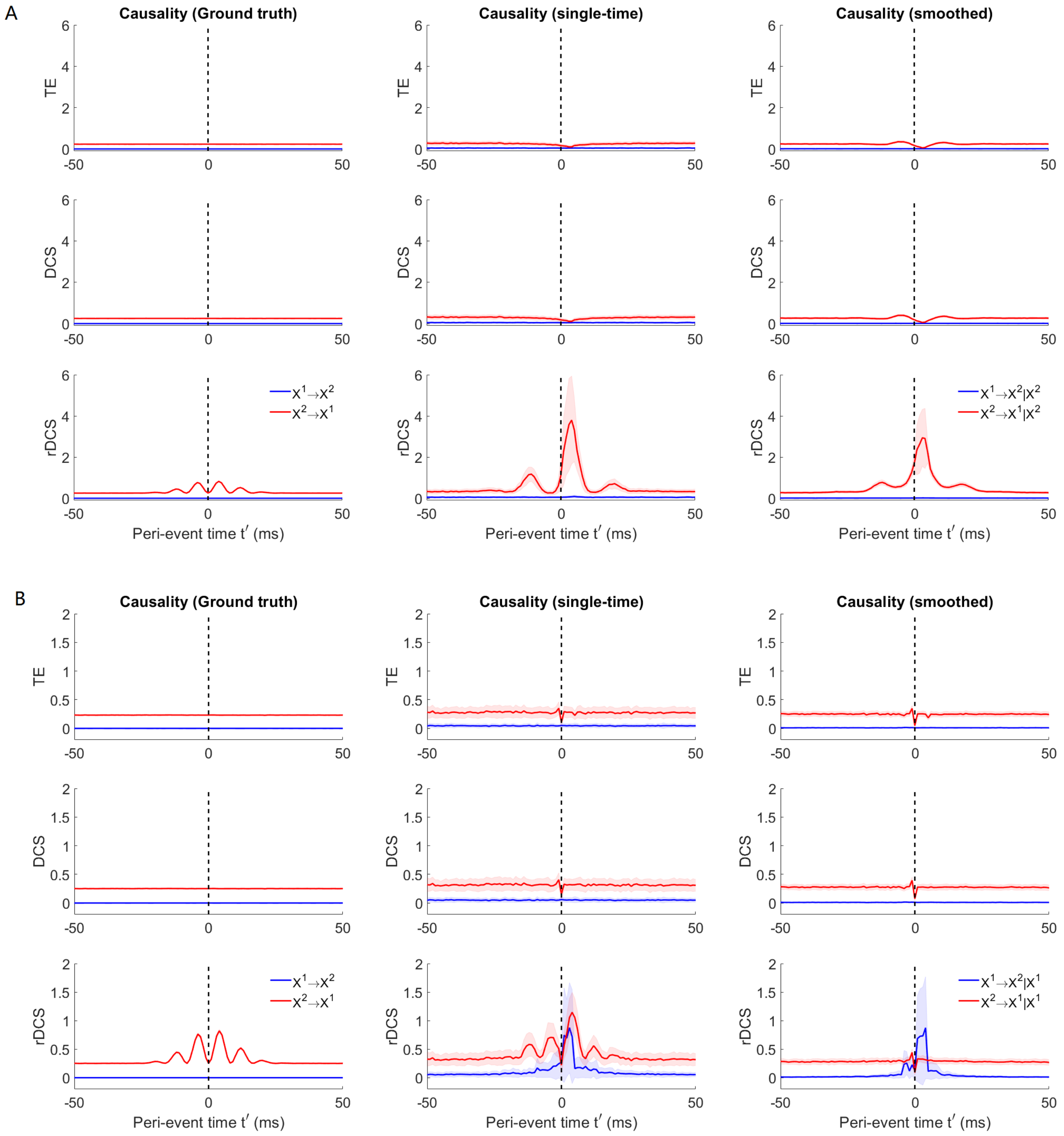

Figure S7: Comparison of causal analysis with different alignments. (A) TE (left), DCS (middle) and rDCS (right) for all 3 types of event ensembles aligned by true causes. (B) TE (left), DCS (middle) and rDCS (right) for all 3 types of event ensembles aligned by true effects.

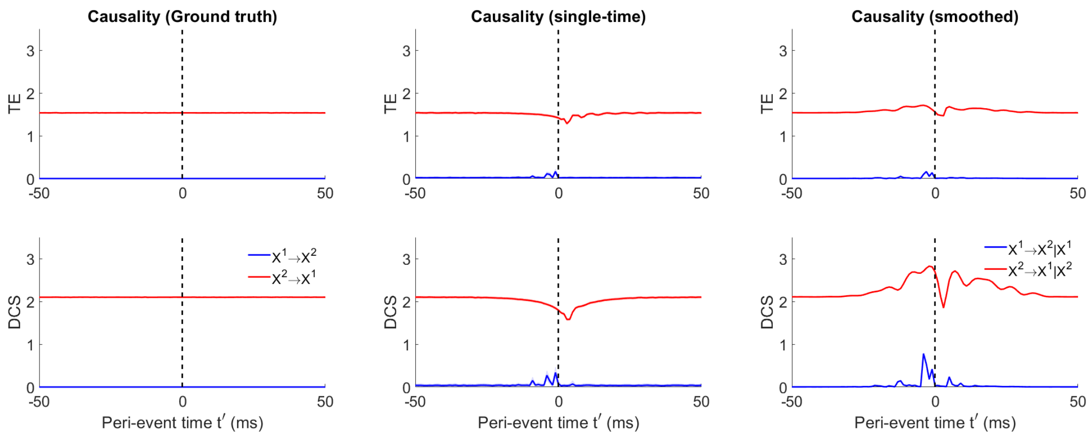

Figure S8: Zoomed TE (upper) and DCS (lower) estimation for 3 types of event ensembles aligned by putative cause.

REFERENCES

Bareinboim, E. and Pearl, J. (2012). Controlling selection bias in causal inference. In *Artificial Intelligence and Statistics*. 100–108

Bareinboim, E., Tian, J., and Pearl, J. (2014). Recovering from selection bias in causal and statistical inference. In *AAAI*. 2410–2416

Bishop, C. M. (2006). *Pattern Recognition and Machine Learning (Information Science and Statistics)* (Berlin, Heidelberg: Springer-Verlag)

Hernán, M. A., Hernández-Díaz, S., and Robins, J. M. (2004). A structural approach to selection bias. *Epidemiology* 15, 615–625

Horwitz, R. I. and Feinstein, A. R. (1978). Alternative analytic methods for case-control studies of estrogens and endometrial cancer. *New England Journal of Medicine* 299, 1089–1094

Janzing, D., Balduzzi, D., Grosse-Wentrup, M., and Schölkopf, B. (2013). Quantifying causal influences. *Ann. Statist.* 41, 2324–2358. doi:10.1214/13-{AOS1145}

Pearl, J. (2000). *Causality: models, reasoning and inference*, vol. 29 (Cambridge Univ Press)

Peters, J., Janzing, D., and Schölkopf, B. (2017). *Elements of Causal Inference – Foundations and Learning Algorithms* (MIT Press)
